# The Glutathione-S-Transferase, Cytochrome P450 and Carboxyl/Cholinesterase Gene Superfamilies in Predatory Mite *Metaseiulus occidentalis*

**DOI:** 10.1371/journal.pone.0160009

**Published:** 2016-07-28

**Authors:** Ke Wu, Marjorie A. Hoy

**Affiliations:** Department of Entomology and Nematology, PO Box 11620, University of Florida, Gainesville, FL 32611, United States of America; Utah State University, UNITED STATES

## Abstract

Pesticide-resistant populations of the predatory mite *Metaseiulus* (= *Typhlodromus* or *Galendromus) occidentalis* (Arthropoda: Chelicerata: Acari: Phytoseiidae) have been used in the biological control of pest mites such as phytophagous *Tetranychus urticae*. However, the pesticide resistance mechanisms in *M*. *occidentalis* remain largely unknown. In other arthropods, members of the glutathione-S-transferase (GST), cytochrome P450 (CYP) and carboxyl/cholinesterase (CCE) gene superfamilies are involved in the diverse biological pathways such as the metabolism of xenobiotics (e.g. pesticides) in addition to hormonal and chemosensory processes. In the current study, we report the identification and initial characterization of 123 genes in the GST, CYP and CCE superfamilies in the recently sequenced *M*. *occidentalis* genome. The gene count represents a reduction of 35% compared to *T*. *urticae*. The distribution of genes in the GST and CCE superfamilies in *M*. *occidentalis* differs significantly from those of insects and resembles that of *T*. *urticae*. Specifically, we report the presence of the Mu class GSTs, and the J’ and J” clade CCEs that, within the Arthropoda, appear unique to Acari. Interestingly, the majority of CCEs in the J’ and J” clades contain a catalytic triad, suggesting that they are catalytically active. They likely represent two Acari-specific CCE clades that may participate in detoxification of xenobiotics. The current study of genes in these superfamilies provides preliminary insights into the potential molecular components that may be involved in pesticide metabolism as well as hormonal/chemosensory processes in the agriculturally important *M*. *occidentalis*.

## Introduction

The predatory mite *Metaseiulus* (= *Typhlodromus* or *Galendromus*) *occidentalis* (Arthropoda: Chelicerata: Arachnida: Acari: Parasitiformes: Phytoseiidae) is an important natural enemy of agricultural pests such as plant-feeding mites in the families Tetranychidae, Tarsonemidae, Eriophyidae and Tenuipalpidae in the USA, Australia, and New Zealand [1–6]. Pesticide-resistant (carbaryl, organophosphate, and sulfur) strains of *M*. *occidentalis* have been developed through laboratory selection and these genetically improved mites have been used in biological control programs [7–9].

The proteins and molecular pathways involved in pesticide resistance in this predator remain undetermined. To confer resistance phenotype, arthropods may employ toxicodynamic changes (e.g. altered target sites that make target proteins less sensitive to pesticides) and/or toxicokinetic modifications (e.g. increased metabolism, decreased penetration, sequestration or increased secretion) [10–13].

The molecular target for sulfur toxicity is unknown in arthropods. In contrast, acetylcholinesterase (AChE) has been identified as the target for carbamate and organophosphate (OP) toxicity. AChE is a key enzyme involved in the degradation of acetylcholine, an important neurotransmitter in both the central and peripheral nervous systems in animals. Carbamates and OPs inhibit AChE activities in arthropods, disrupting nerve impulses, killing these animals or interfering with their ability to carry out normal functions [14]. Arthropod strains resistant to carbamates and OPs may carry an AChE with point mutations (i.e. toxicodynamic changes) that make it less sensitive to these pesticides [10, 12, 15–21]. Alternatively, increased tolerance to OPs can be achieved by elevating the expression of AChE through gene duplication, such as the cases in some resistant strains of *T*. *urticae* [22, 23].

There are many examples of toxicokinetic changes that may contribute to pesticide resistance in arthropods. In insects, numerous studies, including many employing functional expression assays, have shown resistance to pesticides (e.g. carbamates/OPs, DDT, pyrethroids, neonicotinoids and insect growth regulators) can be achieved through elevated expression of pesticide-metabolizing enzymes such as members of the glutathione-S-transferases (GST), cytochrome P450 (CYP), or carboxyl/cholinesterases (CCE) superfamilies [24–39]. Many members of these superfamilies are also involved in other biological processes such as the deactivation of kairomones and pheromones, and biosynthesis of hormones [40–45].

These detoxification enzymes have been studied for their involvement in pesticide metabolism in the Acari as well (for reviews, see [10, 12, 13]). For example, increased activities or expression in GSTs, CYPs and CCEs have been found in *T*. *urticae*, European red mite *Panonychus ulmi*, the scabies mite *Sarcoptes scabiei*, hard ticks *Rhipicephalus bursa*, and the phytoseiid *Phytoseiulus persimilis* that are resistant to pesticides such as pyrethroids, spirodiclofen, tebufenpyrad, abamectin, and etoxazole [31, 46–50]. Other studies have shown that individual members of a particular gene family may be involved in pesticide resistance in Acari, including a CYP gene involved in methidathion resistance in the phytoseiid mite *Amblyseius womersleyi* [51]. GSTs were implicated in permethrin detoxification in scabies mites [52]. High levels of oxidative detoxification were found in *M*. *occidentalis* strains resistant to carbamates, suggesting that members of the CYP superfamily likely play a role in resistance to this group of pesticide [53].

Several recent studies involving functional expression of potential detoxification enzymes provide further details to the pesticide resistance mechanisms in *T*. *urticae*. Two studies showed that two CYP paralogs, CYP392A16 and CYP392E10, were involved in resistance to abmectin and spirodiclofen, respectively [54, 55]. Another study using both functional expression assays and transgenic *Drosophila* experiments showed that CYP392A11 was involved in resistance to acaricides cyenopyrafen and fenpyroximate [56]. Finally, a functional expression study showed that two Delta class and one Mu class GSTs were involved in pesticide resistance [57]. These detailed studies of resistance mechanisms at the molecular level were made possible after the genes encoding these enzymes had been identified through methods such as genomic sequencing.

Genomic sequencing and annotation have been increasingly used to identify large numbers of molecular components that may participate in pesticide resistance process. Subsequent phylogenetic studies may shed light on the evolution and phylogenetic attributes of these gene families that may have functional implications. The GST, CYP and CCE gene superfamilies in *T*. *urticae* are well studied and possess many distinct characteristics such as lineage-specific radiations [58]. For example, while absent in insects, the Mu class GSTs are found in *T*. *urticae* (and tick *Ixodes scapularis*), suggesting they may be Acari-specific within arthropods [58, 59]. In the *T*. *urticae* CCE superfamily, close to the root of neuro/developmental class of CCEs, there are two large new clades (J’ and J”) that are absent in insects [58]. Finally, the majority of the CYPs in *T*. *urticae* form tightly clustered and shallow branches in their phylogenetic trees, suggesting they likely derived from recent gene expansions [13, 58].

Mites and ticks belong to the Chelicerata subphylum of arthropods, and *Metaseiulus occidentalis* separated from *T*. *urticae* approximately 400 million years ago [60]. Little is known about the genes in the GST, CYP and CCE superfamilies in *M*. *occidentalis*, or in any other mite in the Phytoseiidae. In the current study, we manually annotated the GST, CYP and CCE gene complements based on a recently sequenced *M*. *occidentalis* genome [61]. We created phylogenies of these superfamilies. Based on the results of the phylogenetic analyses, we report that *M*. *occidentalis* and *T*. *urticae* share similar patterns of gene distribution in the GST and CCE, and, to a lesser extent, the CYP superfamilies. *Metaseiulus occidentalis* contains fewer genes in all three superfamilies than *T*. *urticae*.

## Materials and Methods

### Manual annotation

To manually annotate *M*. *occidentalis* GST genes, tBLASTn searches were performed on a sequenced *M*. *occidentalis* genome using the GST protein sequences from *D*. *melanogaster*, *Apis mellifera*, *T*. *urticae* and *Homo sapiens* as queries. Gene models were created on the basis of homology and available RNA seq support [62] and were manually assembled in Notepad++. Iterative searches were conducted with each new *M*. *occidentalis* protein as query until no new genes were identified in each major family. The CYP and CCE gene models were assembled in the same manner as GST’s after using the CYP and CCE protein sequences, respectively, from *D*. *melanogaster*, *A*. *mellifera* and *T*. *urticae* as queries to perform tBLASTn searches.

All manually annotated GST, CYP and CCE gene models were initially verified by performing reciprocal BLASTp searches against databases from which query sequences were derived. For further validation, these gene models were used to search the conserved domain database (CDD: http://www.ncbi.nlm.nih.gov/Structure/cdd/wrpsb.cgi) to ascertain that they contain the canonical domains for each type of protein (Domain ID for GSTs: COG0625, PTZ00057, PLN02395, or cd0302; Domain ID for CYPs: pfam00067; Domain ID for CCEs: pfam00135). For the CYP gene models, additional verification was performed by searching for the presence of a CYP signature motif, FXXGXXXCXG, in the heme-binding domain [63, 64]. All CYP sequences were submitted to the cytochrome P450 nomenclature committee (David Nelson, Univ. Tennessee) for naming [65].

To identify the putative catalytically active CCEs, the presence of the esterase-specific catalytic triad Ser-Glu (Asp)-His and the nucleophilic elbow surrounding the active-site serine residue (GXSXG) were examined using criteria described previously [66–68].

### Phylogenetic analyses

For phylogenetic analyses, only the amino acid sequences of the putative full-length GST, CYP and CCE genes (including some probable pseudogenes. For details, see Results and Discussion section), but not those that are clearly pseudogenes or gene fragments, were included. To perform multiple sequence alignments of GSTs, the amino acid sequences of 13 *M*. *occidentalis* cytosolic GST proteins and those of selected homologs from *D*. *melanogaster*, *Anopheles gambiae*, *A*. *mellifera*, *T*. *urticae*, *and I*. *scapularis* were aligned using MAFFT 7.147 with the E-INS-i alignment algorithm and the Blosum62 matrix [69]. For multiple sequence alignments of CYPs, amino acid sequences of 63 *M*. *occidentalis* CYP proteins and those of selected homologs from *D*. *melanogaster*, *A*. *mellifera*, and *T*. *urticae* were aligned. For multiple sequence alignments of CCEs, amino acid sequences of the 44 *M*. *occidentalis* CCE proteins and those of selected homologs from *D*. *melanogaster*, *A*. *mellifera*, and *T*. *urticae* were aligned and the resulting alignment was trimmed at both ends according to the parameters set previously [67].

Phylogenetic analyses were conducted for GSTs, CYPs and CCEs with Bayesian inference using MrBayes v3.2.2 [70]. Model selections were performed with ProtTest 3.2 and the optimum models also supported by MrBayes were selected [71]. According to the Akaike information criterion, the WAG + I + G + F model was selected for the phylogenetic analyses of GSTs and CCEs, and the Blosum62 + I + G + F model was selected for the phylogenetic analyses of CYPs. Metropolis-coupled Markov chain Monte Carlo sampling was performed with one cold and three heated chains. Starting trees were random and the analyses were performed for five million, thirteen million and fourteen million generations for GSTs, CYPs and CCEs, respectively. Samplings were performed every 100 generations. The initial 25% of trees represented burn-in and the remaining trees were used to calculate Bayesian posterior probabilities. The analyses were performed until the average standard deviation of split frequencies dropped below 0.01.

## Results and Discussion

### Glutathione-S-Transferases

Manual annotation of the GST genes in the *M*. *occidentalis* genome produced 16 putative full-length gene models (S1 Table and S1 Fig). These models are identical to the existing GST Gnomon models predicted by the NCBI’s Eukaryotic Genome Annotation Pipeline. Two (Gst1 and GstO1) of the 16 putative full-length gene models contain a partial GST C-terminal domain, suggesting that they are probably pseudogenes. BLASTp searches using the 16 *M*. *occidentalis* GSTs against the GenBank database revealed that GstK1 and GstK2 showed very high similarities (E value = 3e-61and 5e-59 for GstK1 and GstK2, respectively) to a *Homo sapiens* Kappa class GST (GenBank accession number: NP_057001.1), which is a mitochondrial GST. A *M*. *occidentalis* GST (PTGSES2) showed a high degree of similarity (E value = 2e-87) to a *H*. *sapiens* microsomal GST (GenBank accession number: JC7977), a membrane-associated prostaglandin E synthase-2. The Kappa class GSTs belong to an ancient family of proteins with orthologs in bacteria and eukaryotes that may play a role in detoxification, energy and lipid metabolism, and may even act as chaperones to facilitate correct folding and assembly of proteins [72]. The microsomal GSTs may play a role in protection against oxidative stress and disarming toxic xenobiotics [73]. It is possible that the Kappa and microsomal class GSTs in *M*. *occidentalis* serve similar functions as their homologs in other species.

**Fig 1:**
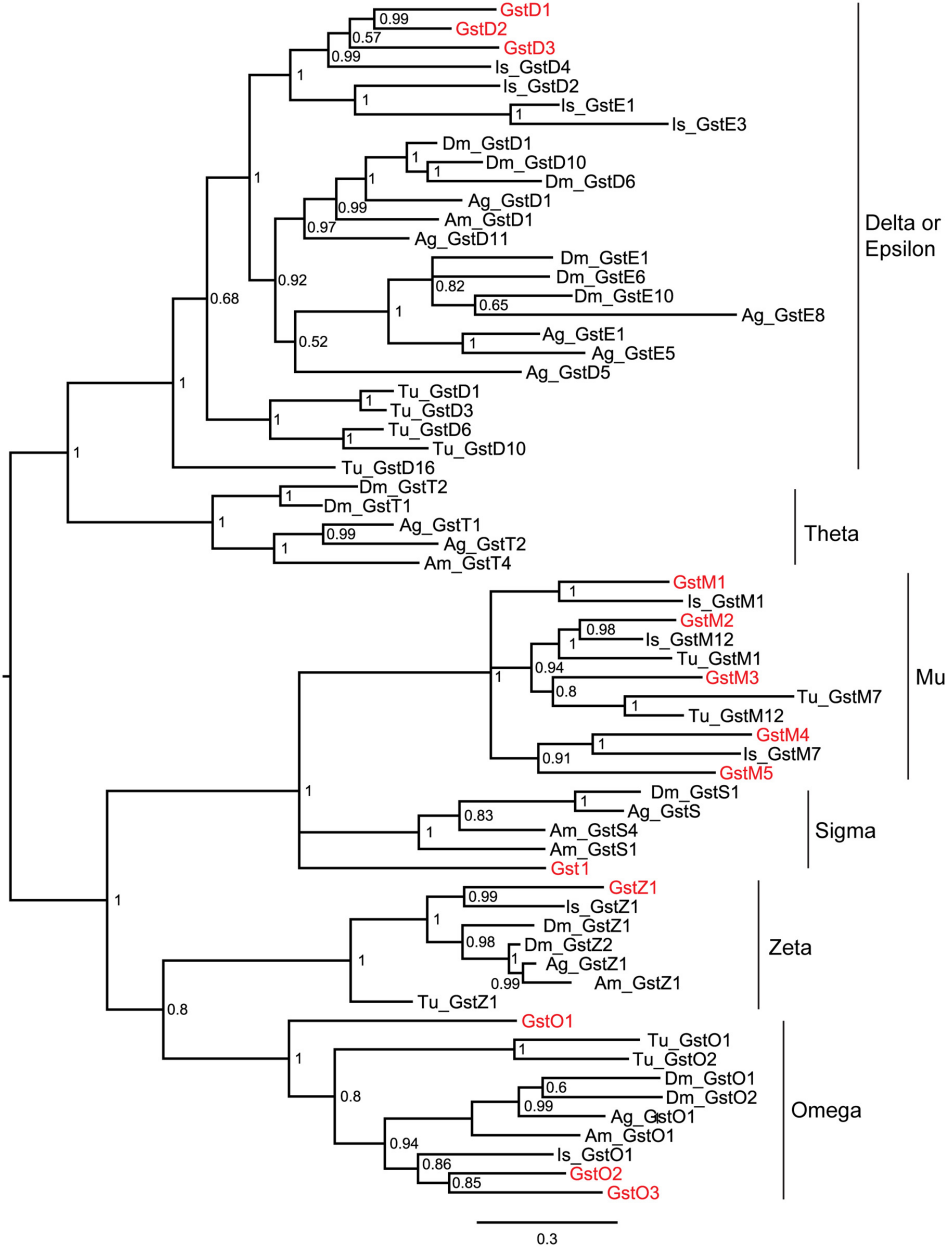
Phylogenetic relationships of the different cytosolic GST classes. The deduced amino acid sequences of 13 *M*. *occidentalis* cytosolic GST genes were aligned with those of selected GSTs from *D*. *melanogaster* (Dm), *An*. *gambiae* (Ag), *A*. *mellifera* (Am), *T*. *urticae* (Tu) and *I*. *scapularis* (Is) (S2 Fig). The midpoint-rooted tree was generated using MrBayes. The *M*. *occidentalis* GST genes are shown in red. Posterior probabilities are shown at the nodes. Details of the gene names for the GSTs from *M*. *occidentalis* and other arthropods are shown in S1 and S2 Tables, respectively.

The rest of the *M*. *occidentalis* GSTs belong to different cytosolic classes (Fig 1 and Table 1). The number of the cytosolic GST genes (13) is far fewer than those in *T*. *urticae* (31) and *I*. *scapularis* (32), the two other acarine species included in the current comparison (Table 1). Among the nine arthropods listed, *M*. *occidentalis* has the second fewest cytosolic GSTs, ranked only above the honey bee *A*. *mellifera*, which has 8 (Table 1).

**Table 1:** A comparison of cytosolic GST gene numbers in the genomes of nine arthropods. Data are derived from Hayes et al. [73], Oakeshott et al. [84], Grbic et al. [58], Reddy et al. [59], and the current study.

GST family	*D*. *melanogaster*	*An*. *gambiae*	*T*. *castaneum*	*B*. *mori*	*A*. *mellifera*	*N*. *vitripennis*	*T*. *urticae*	*I*. *scapularis*	*M*. *occidentalis*
Delta/Epsilon	25	20	22	12	1	5	16	12	3
Mu	0	0	0	0	0	0	12	14	5
Omega	5	1	4	4	1	2	2	3	3
Sigma	1	1	7	2	4	8	0	0	0
Theta	4	2	1	1	1	3	0	0	0
Zeta	2	1	1	2	1	1	1	3	1
Unknown	0	3	0	2	0	0	0	0	1
Total	37	28	35	23	8	19	31	32	13

Phylogenetic analyses of the *M*. *occidentalis* cytosolic GSTs revealed four different classes of GSTs: Delta/Epsilon (3 genes), Mu (5 genes), Omega (3 genes) and Zeta (1 gene) (Fig 1). GST1 does not cluster with any known class. A comparison of the cytosolic GST distribution in different classes in *M*. *occidentalis* vs. the other eight arthropods revealed some interesting findings (Table 1). All nine arthropods contain the Delta/Epsilon, Omega and Zeta class GSTs. Only Acari contain homologs that are similar to the mammalian Mu class GSTs (Table 1) [59]. As in *I*. *scapularis*, the Mu class is the largest GST class in *M*. *occidentalis*. In general, the numbers of the Mu class GSTs in all three acarine species are comparable to those of the Delta/Epsilon classes. Finally, unlike insects, all three acarine species lack the Sigma or Theta class GSTs. The functions of the Sigma and Theta class GSTs remain poorly understood, with some studies suggesting that the Sigma class GSTs may be involved in protection against oxidative stress [74–76]. The implications for the lack of the Sigma and Theta class GSTs in Acari remain unclear.

The ubiquitous distribution of the Delta/Epsilon, Omega and Zeta class GSTs in arthropods suggests that they play important roles in endogenous metabolic processes. Members of the Delta/Epsilon class of GSTs are directly involved in pesticide resistance in mosquito disease vectors and *T*. *urticae* [28, 29, 57, 77]. The Omega class GSTs are involved in the removal of S-thiol adducts from proteins [78]. The Zeta class GSTs catalyze the degradation of tyrosine and phenylalanine and may also be involved in pesticide resistance [79, 80]. The results from two recent studies indicate the Omega and Zeta class GSTs may also be involved in oxidative stress response [81, 82]. Similarly, the mammalian Mu class GSTs have been suggested to participate in oxidative stress response, which may be caused by insecticides [83]. A recent study in *T*. *urticae* showed that a Mu class GST was involved in pesticide resistance [57]. The *M*. *occidentalis* GST homologs in these classes may play a similar role as their counterparts in other species.

**Fig 2:**
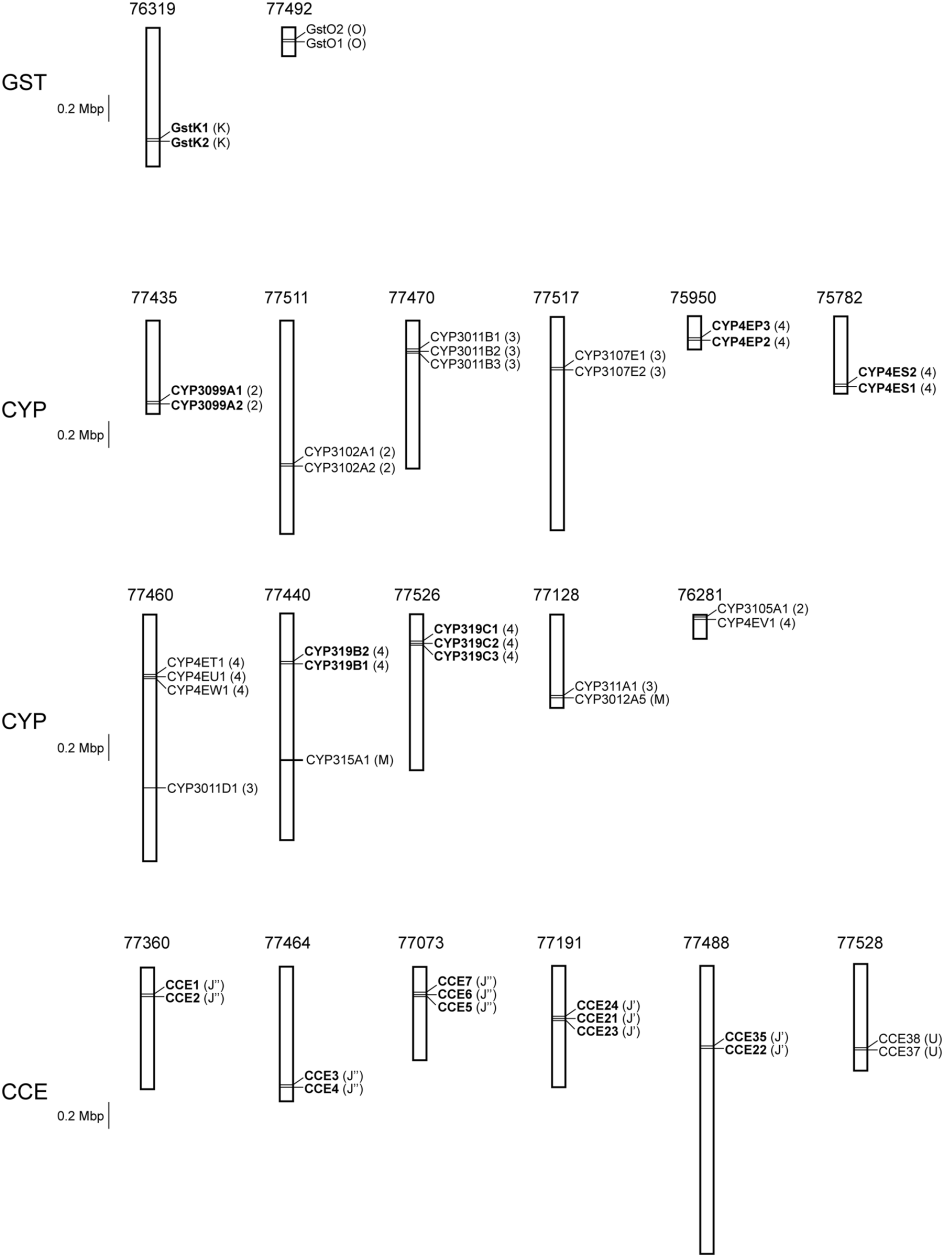
A schematic diagram of the GST, CYP and CCE tandem arrays and gene clusters on *M*. *occidentalis* genome scaffolds. Each scaffold is represented by a bar with its ID (prefix is scf71800000) indicated on top. Scaffold sequences are numbered from top (first nucleotide) to bottom. The names of the genes present in tandem are highlighted in bold. The GST class (K and O = Kappa and Omega classes, respectively), CYP clan (2, 3, 4 and M = Clans 2, 3, 4 and M, respectively) and CCE clade assignments (J’, J” and K = Clades J’, J” and K, respectively; U = Undermined) are shown in brackets.

Similar to the GST genes in other sequenced arthropod genomes [84], the *M*. *occidentalis* GST genes show some clustering in their genomic locations (Fig 2). Both Kappa class GSTs are located in a tandem array and two of three Omega class GSTs (GstO2 and GstO3) form a cluster (Fig 2). The cluster and tandem array are likely the result of gene duplication.

### Cytochrome P450s

A total of 75 CYP gene models were produced by manual annotation, 12 of which are apparent pseudogenes as determined by the cytochrome P450 nomenclature committee. Among the 63 putative full-length CYP gene models, six appear to contain an incomplete CYP domain and may also be pseudogenes (S1 Table and S1 Fig). Unlike the *M*. *occidentalis* GSTs, not all *M*. *occidentalis* CYP Gnomon models appear to be correct. While some Gnomon models appear to have concatenated multiple genes, others appear to have been poorly assembled. Among the 63 putative full-length CYP gene models, 12 represent novel gene models or improved Gnomon gene models and the rest are identical to the existing Gnomon models (S1 Table and S1 Fig). The lower proportion of correct CYP (also CCE, see below) Gnomon gene model predictions likely reflects the fact that CYPs (also CCEs), when compared to GSTs, are less conserved across species. Our annotation result is consistent with the notion that manual annotation is needed to achieve a more accurate CYP gene assembly [85].

**Fig 3:**
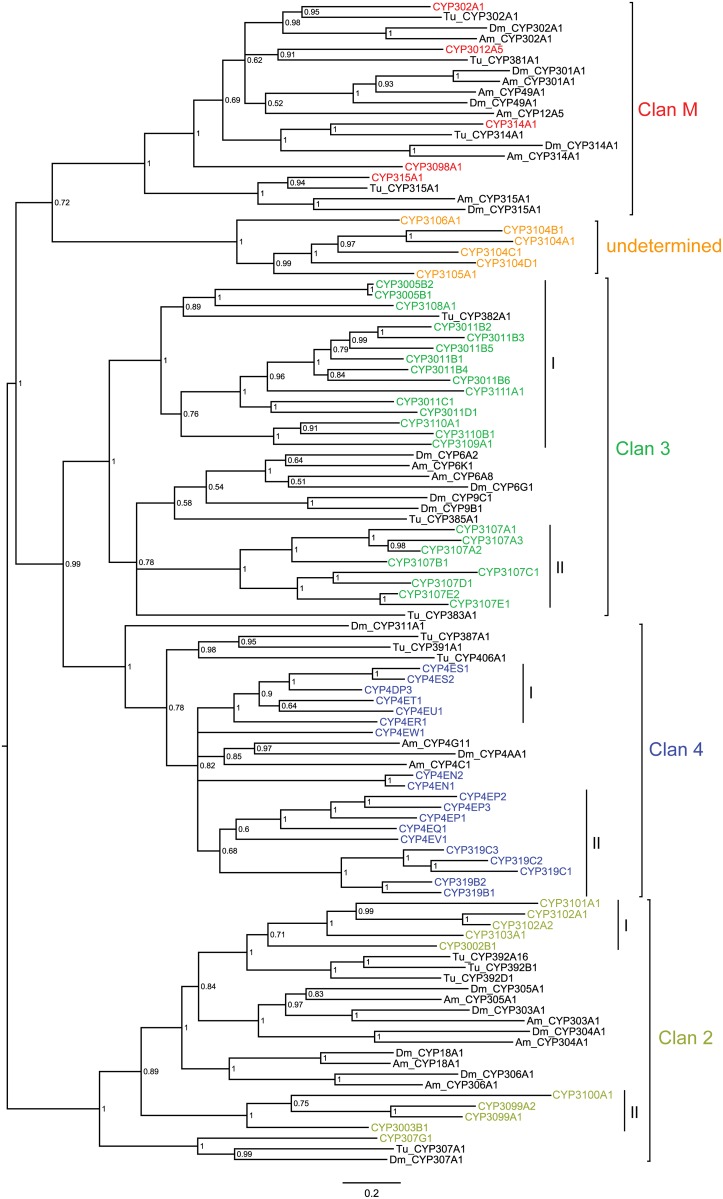
Phylogenetic relationships of the different CYP clans. The deduced amino acid sequences of 63 *M*. *occidentalis* CYP genes were aligned with those of selected CYPs from *D*. *melanogaster* (Dm), *A*. *mellifera* (Am) and *T*. *urticae* (Tu) (S3 Fig). The midpoint-rooted tree was generated using MrBayes. The *M*. *occidentalis* CYP genes are shown in colors. Posterior probabilities are shown at the nodes. Details of the gene names for the CYPs from *M*. *occidentalis* and other arthropods are shown in S1 and S3 Tables, respectively.

The number of the CYP genes in *M*. *occidentalis* is fewer than those in *T*. *urticae* (86) and other arthropods listed (Table 2), with the exception of *A*. *mellifera* (46). CYPs in arthropods can be grouped into four distinct clans: CYP2, CYP3, CYP4 and the mitochondrial clan, based on sequence similarities and phylogenies [86]. Assignment of the *M*. *occidentalis* CYP genes into different clans, families, and subfamilies was initially achieved on the basis of sequence similarity by the P450 nomenclature committee, and was further supported, with a few exceptions, by subsequent phylogenetic analyses (Fig 3) [65]. The distribution of the *M*. *occidentalis* CYPs in various clans differs from that of *T*. *urticae*, and instead resembles those of insects (Table 2). The CYP3 (23 genes) is the most abundant clan in *M*. *occidentalis*, followed by the CYP4 (19 genes) and CYP2 (16 genes) clans. The mini-blooms in *M*. *occidentalis* CYP3s were also reported by Van Leeuwen and Dermauw when performing a phylogenetic study using CYP Gnomon gene models [2016]. By comparison, there is a huge expansion of the CYP2 clan (48 genes) and an equally dramatic reduction of the CYP3 clan (10 genes) in *T*. *urticae* [58].

**Table 2:** A comparison of CYP gene numbers in the genomes of eight arthropods. Data are derived from Feyereisen [25], Grbic et al. [58] and the current study.

CYP clan	*D*. *melanogaster*	*An*. *gambiae*	*T*. *castaneum*	*B*. *mori*	*A*. *mellifera*	*N*. *vitripennis*	*T*. *urticae*	*M*. *occidentalis*
CYP2	7	10	8	7	8	7	48	16
CYP3	36	40	72	30	28	48	10	23
CYP4	32	46	45	36	4	30	23	19
Mitochondrial CYP	11	9	9	12	6	7	5	5
Total	88	105	134	85	46	92	86	63

The *M*. *occidentalis* mitochondrial CYP clan contains five genes, three of which show 1:1:1:1 orthologies with the mitochondrial CYPs from other arthropod genomes included in our phylogenetic analyses (*T*. *urticae*, *A*. *mellifera* and *D*. *melanogaster;* Fig 3). CYP302A1, CYP314A1 and CYP315A1 are orthologs of enzymes encoded by *D*. *melanogaster* Halloween genes *disembodied* (Dm_CYP302A1 in tree), *shade* (Dm_CYP314A1 in tree), and *shadow* (Dm_CYP315A1 in tree), respectively. These *D*. *melanogaster* genes encode for steroid hydroxylases that are involved in the biosynthesis of insect molting hormone, 20-hydroxyecdysone (20E) [43, 44]. It is likely that *M*. *occidentalis* orthologs perform similar functions. The *M*. *occidentalis* CYP3012A5 and CYP3098A1 genes do not appear to have orthologs in insects.

In the *M*. *occidentalis* CYP2 clan, CYP307G1 appears to be an ortholog of CYP307A1 of *D*. *melanogaster* and *T*. *urticae* (Fig 3). *Drosophila melanogaster* CYP307A1 encodes for *spook*, which is required for the biosynthesis of 20E [44]. It is likely that CYP307G1 is involved in 20E biosynthesis in *M*. *occidentalis*. The rest of the *M*. *occidentalis* CYP2s belong to two tightly clustered clades that share no strong orthology with CYP2s from the other arthropods evaluated, suggesting they might have derived from recent gene expansion events (Fig 3). However, it is possible that potential orthologs from other species may be present in the sequences not included in the current study. Clade I contains CYP3101A1, CYP3102A1, CYP3102A2, CYP3103A1 and CYP3002B1. They cluster with members of the *T*. *urticae* CYP392 family, some of which (e.g. CYP392A16 in tree) are be involved in the resistance to several pesticides [54–56]. Members of the clade II of the *M*. *occidentalis* CYP2 clan do not share close sequence similarities with CYP2s from *D*. *melanogaster*, *A*. *mellifera*, or *T*. *urticae* (Fig 3). Six CYPs (CYP3106A1, CYP3104B1, CYP3104A1, CYP3104C1, CYP3104D1, and CYP3105A1) could not be assigned to any of the four CYP clans by Bayesian analyses (Fig 3). This is not surprising because the classification of some CYP genes can be difficult, due to significant differences in their sequences among species [86]. They were, however, assigned to the CYP2 clan based on sequence identities by the CYP nomenclature committee (S1 Table).

Interestingly, similar to *T*. *urticae*, *M*. *occidentalis* also lack orthologs to CYP306A1 and CYP18A1, two genes that encode, respectively, the biosynthetic C25 hydroxylase and a C26 hydroxylase/oxidase involved in hormone inactivation. These results suggest that *M*. *occidentalis* may use alternative enzymes in place of these two CYPs. Alternatively, this predatory mite may use a different molting hormone (e.g. ponasterone A) instead of 20E, as suggested in *T*. *urticae* [58].

Insect CYPs usually lack precise orthologies in the CYP3 clan [84]. Unsurprisingly, members of the *M*. *occidentalis* CYP3 clan do not show close sequence similarities with insect CYP3s. *Metaseiulus occidentalis* CYP3s are separated into two clades consisting of 15 and 8 genes each. They diverge from the insect CYP6 and 9 families (Fig 3). It is notable that the insect CYP6 family contains members that are involved in resistance to a broad range of chemically unrelated pesticides [25, 87] and the detoxification of host plant secondary metabolites in the gut [26, 88].

The majority of the *M*. *occidentalis* CYP4 clan can be divided into two clades that consist of 6 and 10 genes each (Fig 3). CYP4EW1, CYP4EN1 and CYP4EN2 could not be assigned to either clade due to polytomy. None of the genes in *M*. *occidentalis* CYP4 clan show precise orthologies with the selected CYP4s from either insects or the spider mite. The insect CYP4 clan comprises highly diverse families of enzymes that have been implicated in the metabolism of insecticides and pheromone perception [89–92]. The specific functions of *M*. *occidentalis* CYP4s remain to be determined.

Our CYP annotation and phylogenetic analysis results are consistent with the notion that arthropod CYPomes consist of many species- or lineage-specific expansions of CYP subfamilies and only a small number of recognizable orthologs [93]. Many CYP gene expansions in *M*. *occidentalis* appear to have resulted from recent gene duplication events, as evidenced by the physical clustering of the CYP genes on genomic scaffolds (Fig 2). Eleven CYPs are present in small tandem arrays of 2–3 genes each. The numbers of these tandem repeats are small when compared to those in some other arthropods such as *Daphnia pulex* [94]. Fourteen CYPs form gene clusters composing of 2–3 genes. As found in other arthropods, with very few exceptions, CYPs in the same tandem array and physical cluster are also from the same phylogenetic radiation (Figs 2 and 3) [84, 94].

### Carboxyl/cholinesterases

The *M*. *occidentalis* genome contains 44 full-length CCE genes and one apparent pseudogene. Of the 44 putative full-length genes, three contain a partial esterase domain and may be pseudogenes (S1 Table). Thirty four of 45 gene models are identical to the existing Gnomon models. The rest (11) represent either new gene models or refined Gnomon gene models (S1 Table and S1 Fig). The total number of CCEs in *M*. *occidentalis* is similar to that of the wasp *Nasonia vitripennis* and much fewer than the 71 CCEs found in *T*. *urticae* (Table 3).

**Table 3:** A comparison of CCE gene numbers in the genomes of eight arthropods. Data are derived from Yu et al.[106], Oakeshott et al [84]. Grbic et al [58]. and the current study.

CCE clade	*D*. *melanogaster*	*An*. *gambiae*	*T*. *castaneum*	*B*. *mori*	*A*. *mellifera*	*N*. *vitripennis*	*T*. *urticae*	*M*. *occidentalis*
**Dietary/detoxification class**								
Clade A, B and C	13	16	26	57	8	13	0	0
**Hormone/semiochemical class**								
Clade D (integument esterases)	3	0	2	2	1	4	0	0
Clade E (secreted β esterases)	2	4	7	2	2	11	0	0
Clade F (dipteran JhE)	3	6	2	4	2	2	0	0
Clade G (lepidopteran JhE)	0	4	0	0	0	0	0	0
Clade F’ (crustacean/Acari JhE)	0	0	0	0	0	0	2	0
**Neuro/developmental class**								
Clade H (glutactin)	5	10	1	1	1	1	2	0
Clade I (uncharacterized clade)	1	1	1	2	1	1	0	0
Clade J (AChEs)	1	2	2	2	2	2	1	1
Clade K (gliotactin)	1	1	1	1	1	1	1	1
Clade L (neuroligins)	4	5	5	3	5	5	5	5
Clade M (neurotactins)	2	2	2	2	1	1	1	0
**Novel Acari-specific class**								
Clade J’	0	0	0	0	0	0	34	19
Clade J”	0	0	0	0	0	0	22	15
Undetermined	0	0	0	0	0	0	3	3
Total	35	51	49	76	24	41	71	44

**Fig 4:**
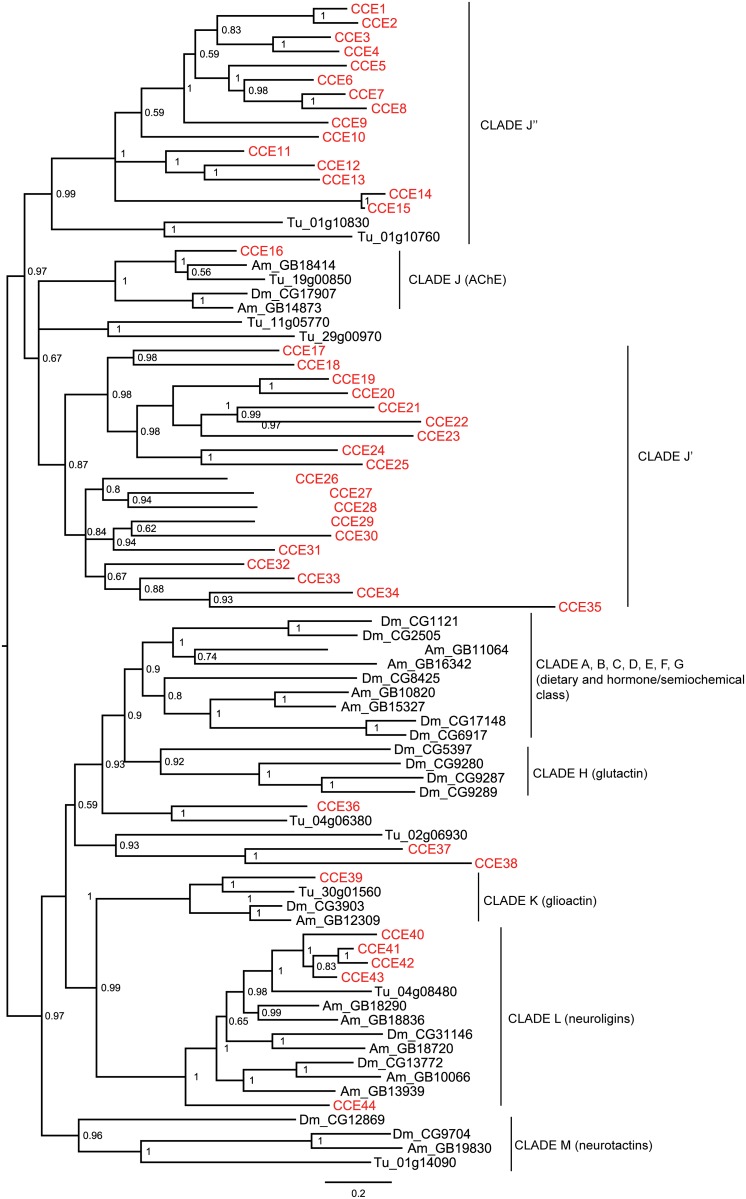
Phylogenetic relationships of the different CCE classes/clades. The deduced amino acid sequences of 44 *M*. *occidentalis* CCE genes were aligned with those of selected CCEs from *D*. *melanogaster* (Dm), *A*. *mellifera* (Am) and *T*. *urticae* (Tu) (S5 Fig). The alignment was trimmed at both ends using criteria set previously [67]. The midpoint-rooted tree was generated using MrBayes. The *M*. *occidentalis* CCE genes are shown in red. Posterior probabilities are shown at the nodes. Details of the gene names for the CCEs from *M*. *occidentalis* and other arthropods are shown in S1 and S4 Tables, respectively.

Insect CCEs fall into three main phylogenetic classes with broadly defined, yet distinct functions: dietary/detoxification, hormone/semiochemical processing and neuro/developmental functions [84]. The phylogenetic relationships among *M*. *occidentalis* CCEs and those from several arthropod species were investigated (Fig 4). The distribution of *M*. *occidentalis* CCEs among different CCE classes/clades, while similar to that of *T*. *urticae*, differs from those of insects in two significant ways (Table 3). First, both *M*. *occidentalis* and *T*. *urticae* lack homologs to insect CCEs in the dietary/detoxification and hormones/semiochemical classes (clades A, B, C, D, E, F and G). Secondly, the majority of *M*. *occidentalis* CCEs fall into two new clades (CCE17–CCE35 in the J’ clade and CCE1–CCE15 in the J” clade) close to the root of the neuro/developmental class of CCEs. This distribution pattern is similar to that of the *T*. *urticae* CCE superfamily, in which 34 and 22 CCEs fall into the clades J’ and J”, respectively [58]. Taken together, these results suggest that the J’ and J” clades likely represent two ancient, Acari-specific clades, although we cannot rule out the possibility that orthologs to *M*. *occidentalis* genes in these clades may exist in the excluded CCE sequences from other species. Interestingly, the majority of the *M*. *occidentalis* CCEs in the J’ (12 out of 19) and J” (12 out of 15) clades contain characteristic features of α/β-hydrolase structure [66–68], such as a catalytic triad composed of Ser-Glu (Asp)-His and the nucleophilic elbow surrounding the active-site serine residue (GXSXG) (S1 Table and S4 Fig), indicating that they are catalytically active. Similarly, three of four *T*. *urticae* CCEs in the J’ and J” clades selected for the phylogenetic analyses also contain the same features (S4 Fig).

The majority of CCEs in insect genomes belong to the dietary/detoxification and hormone/semiochemical classes with each species having representatives in most of the clades in these two classes [84]. Unlike most CCEs in the neuro/developmental class, CCEs in the dietary/detoxification and hormone/semiochemical classes are mostly catalytically active and participate in diverse biological process such as the detoxification of xenobiotics (e.g. insecticide metabolism) and the hormone/pheromone processing [26, 27, 67, 95]. We speculate that CCEs in the J’ and J” clades perform similar functions in *M*. *occidentalis* as those in insect dietary/detoxification or hormone/semiochemical classes due to the presence of the intact catalytic triads and the apparent need for such important biological functions encoded by these classes of CCEs in this mite.

In the neuro/developmental class, *M*. *occidentalis* contains one ortholog of gliotactin and five orthologs of neuroligins (Fig 4). Gliotactin and neuroligins are catalytically inactive cholinesterase-like molecules that are involved in the cell-cell interactions during the development of the nervous system [96–98]. The high degrees of orthologies of these proteins across different arthropods suggest that they likely play conserved functional roles in these species (Table 3). As expected, none of the CCEs in the *M*. *occidentalis* K (gliotactin) or L (neuroligins) clades contains an intact catalytic triad (S1 Table and S4 Fig). Three *M*. *occidentalis* CCEs (CCE36-CCE38) do not cluster with any insect CCE clade (Fig 4). None contain an intact catalytic triad, suggesting that they likely play a structural role (S1 Table and S4 Fig).

The *M*. *occidentalis* genome has one copy of AChE gene (CCE16 in tree, Fig 4). Similar to its orthologs from *D*. *melanogaster*, *A*. *mellifera* and *T*. *urticae*, the *M*. *occidentalis* AChE contains the signature features of an active esterase as described above (S1 Table, S1 and S4 Figs). DNA used for the genome sequencing project was produced from a *M*. *occidentalis* strain that is resistant to carbamates and OPs [61]. The presence of a single copy of AChE gene suggests this mite, unlike some of the OP-resistant *T*. *urticae* strains [22, 23], does not utilize an AChE gene expansion strategy to confer resistance to carbamate/OPs.

**Fig 5:**
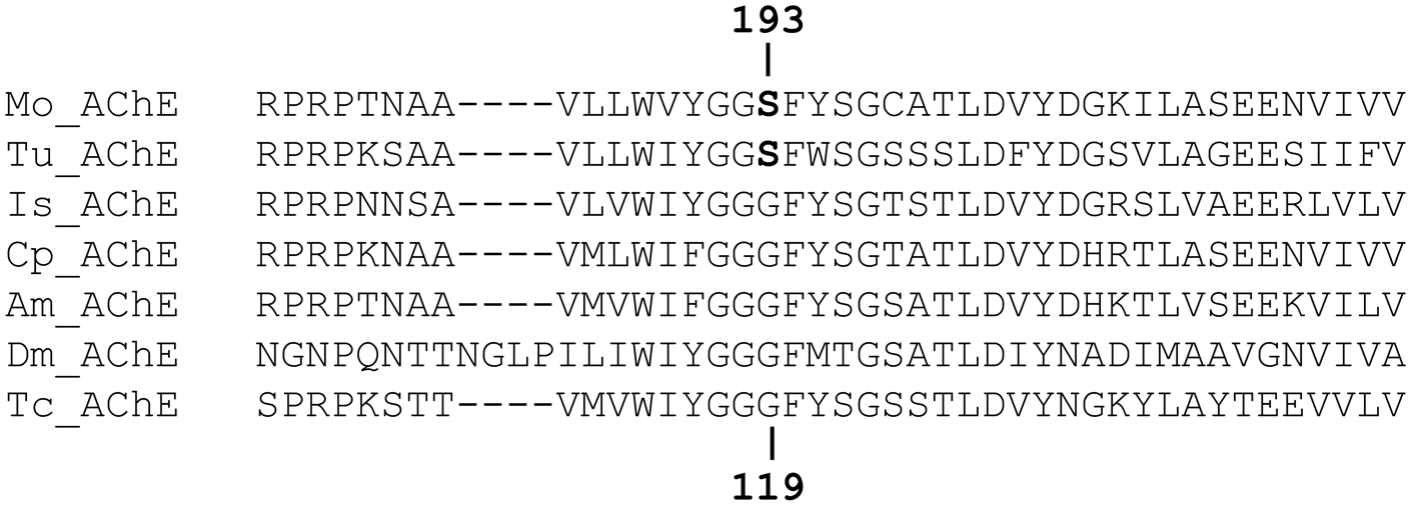
A schematic diagram showing the point mutation at a conserved position in the AChEs of *M*. *occidentalis* and *T*. *urticae*. The deduced amino acid sequences of the AChEs from several species were aligned using the same method as described for other multiple sequence alignments (e.g. GSTs). A partial alignment is shown with the G-to-S point mutation in the *M*. *occidentalis* and *T*. *urticae* AChEs highlighted in bold. Numbers on the top and bottom of the alignment denote the positions of corresponding amino acid residues in the AChEs of *M*. *occidentalis* and *T*. *californica*, respectively. Species include *M*. *occidentalis* (Mo; CCE16 in this study), *T*. *urticae* (Tu; OrcAE ID: tetur19g00850), *I*. *scapularis* (Is; GenBank accession no.: XP_002413212.1), *Culex pipiens* (Cp; GenBank accession no.: AAV28503.1), *A*. *mellifera* (Am; BeeBase ID: GB18414), *D*. *melanogaster* (Dm; FlyBase ID: CG17907) and *T*. *californica* (Tc; UniProtKB ID: P04058.2).

An examination of the deduced amino acid sequence of the *M*. *occidentalis* AChE identified a G(193)S mutation (corresponding to the conserved position 119 of the mature AChE of *Torpedo californica*) that is associated with resistance to propoxur (a carbamate) in *Culex pipiens* and *An*. *gambiae* [15, 99, 100], and chlorpyrifos (an OP) resistance in a closely related predatory mite *Kampimodromus aberrans* (Acari: Phytoseiidae) [21]. Interestingly, the same point mutation is also found in the amino acid sequence of the AChE of *T*. *urticae* (London strain), although it is unclear whether this strain of *T*. *urticae* is resistant to carbamates/OPs (Miodrag Grbic, personal communications). The G119S amino acid substitution is not found in *I*. *scapularis*, several insects or *T*. *californica* (Fig 5). And similar to the AChE in chlorpyrifos-resistant strain of *K*. *aberrans*, no other point mutations associated with carbamate/OP resistance in insects or Acari were found in the *M*. *occidentalis* AChE [12, 15–18, 101]. Future studies are needed to compare the AChE sequences from *M*. *occidentalis* strains that are resistant and susceptible to carbamates/OPs in order to determine whether the G(193)S point mutation (or other possible yet-to-be found point mutations) is associated specifically with the resistance phenotype. If confirmed, follow-up studies involving the *in vitro* expression of the AChEs from susceptible and resistant strains are required to validate the notion that this mutation indeed confers resistance.

Similar to the GST and CYP genes, many CCE genes also exist in physical clusters on genome scaffolds. Twelve CCEs form 5 tandem arrays of 2–3 genes each and two CCEs form 1 gene cluster on *M*. *occidentalis* genome scaffolds (Fig 2). As with GSTs and CYPs, the physical clustering of CCEs also overlaps with the phylogenetic radiation (Figs 2 and 4).

## Conclusions

The current study provides the first glimpses into the shared and unique features of the GST, CYP and CCE gene superfamilies in the phytoseiid mite *M*. *occidentalis* in comparison with other arthropods. *Metaseiulus occidentalis* has fewer members of the GST, CYP and CCE superfamilies than the spider mite *T*. *urticae*. This difference likely reflects a reduced need for detoxification in *M*. *occidentalis*, possibly due to the fact that this obligatory predator is likely exposed to a narrower range of potentially toxic xenobiotics in their prey diet than the plant-feeding spider mite. Clearly, further studies are needed to determine the diverse functions encoded by these genes that our current analyses have inferred. For example, to delineate the molecular mechanisms underlying pesticide resistance, further characterization and comparisons of the expression levels (or in the case of AChEs, amino acid sequences) of these genes in resistant and susceptible strains of *M*. *occidentalis* are required. Results from these future studies, coupled with functional genomic analyses using approaches such as RNAi [102–105], could tease out the molecular mechanisms for pesticide resistances in *M*. *occidentalis*.

## Supporting Information

S1 Fig The nucleotide and deduced amino acid sequences of GST, CYP and CCE gene models (including pseudogenes) of *M*. *occidentalis*. (TXT)

S2 Fig A multiple sequence alignment of selected GST genes. (TXT)

S3 Fig A multiple sequence alignment of selected CYP genes. (TXT)

S4 Fig A multiple sequence alignment of selected CCE genes. (DOCX)

S1 Table Details of the *M*. *occidentalis* GST, CYP, and CCE genes and proteins. (DOCX)

S2 Table Gene ID of the GST sequences from several arthropods used for phylogenetic analyses. (DOCX)

S3 Table Gene ID of the CYP sequences from several arthropods used for phylogenetic analyses. (DOCX)

S4 Table Gene ID of the CCE sequences from several arthropods used for phylogenetic analyses. (DOCX)
