# Supplementary material for: The Glutathione-S-Transferase, Cytochrome P450 and Carboxyl/Cholinesterase Gene Superfamilies in Predatory Mite Metaseiulus occidentalis
Source: PLoS One. 2016 Jul 28;11(7):e0160009. doi: 10.1371/journal.pone.0160009 (PMC4965064; doi:10.1371/journal.pone.0160009)
Supplement: S4 Fig — (DOCX) [file pone.0160009.s004.docx]

**S4 Fig.**

A multiple sequence alignment file (in FASTA format) of the deduced amino acid sequences of the CCE genes from the mites *M. occidentalis* and *T. urticae*, the insects *A*. *mellifera* (Am) and *D. melanogaster* (Dm). Gene details for the CCE genes from *M. occidentalis* and other arthropods are shown in S1 and S4 Tables, respectively. Sequences were trimmed according to Claudianos et al. [1] and do not include N- and C-terminal extensions typical of many neuro/developmental members of this family. The resultant alignment spans a region equivalent to residues 65–558 of D. melanogaster AChE (Dm_CG17907). The characteristic α/β-hydrolase structural features such as the catalytic triad composed of Ser-Glu (Asp)-His (i.e. S-E (D)-H) and the nucleophilic elbow surrounding the active-site serine residue (GXSXG) were identified using criteria described previously [1-3], and are highlighted in red/bold and bold letters, respectively.

>CCE33

NSFLGIPYAQPPVGG--MRFE---------------RPMEFP-P----W--------TGVFEAKKLPKSCPQPTMQL-----NGF-----------------------VGVNV----SDTSEDCLYLNIWSPFCDG------------------------------SKKCRSRP--------------VIVYIHG------GGFIHGGTNWP-IFDGAELAAKAET-VVVSLNYRLGALGFLYVP---------SNGGRPERANMGLYDQHLALRWVKSNIKAFGG-----DHNKITIMGQDAGSASVGYH-ILTP-R---STG--LFKRAVMQSGSPFSFVLRNTKD-QGETLFRS-LASYTDCMS---VHFNS-TLRYDDVLQCMKKQP-FENIIAASDKFN------------GKGANSFFPI---------------MG---------E-----------EFI-PM----NP---------KDS------------------LLLK-------------------RF-------------------------------------------SNVD-LLLSTTKS**E**GAFFL-QHFLSPF---TNVN----DPDKINPGEI-----VFYL-------RVFLSALLGG-------------KP------TASLNELTQNTE--PETR---------EEKI-------QFLKNI-------------------------SAVIGDYPYLCATTEFGTE-------Y-ANL------------------R--HNVYHMQYDHRPWF----LLH---------------P------------TWFP---ST--**H**NDDIMFWL--------GSVYK-LK---------------------ERTRADERVADELIAILTAFASKG

>CCE32

HTFLGVPFAQPPVGK--LRFR---------------KPVPVK-P----W--------LRELKAMFLPPSCAQPDVVV-----NRV-----------------------FNFTS----EERSEDCLYLNIWSPAKNN------------------------------TPDEGPKT--------------VMVYLYG------GAFFFGSTNYR-FYDGAMLAALTDV-VVVSVNYRLGPLGFMNAR---------IP---EIPGNQGLWDQHLAFRWIKENIANFNG-----NPDSITIF**G**H**S**A**G**GISLGFH-LVSP-L---SKG--LFHRAIIQSGAPYYKITDNPEE--ARLQFEK-AATELSCAS---EEDFEGEKDMNEIVDCMLSKN-TSEILTSLEYFN------------DRVKTTYIPW---------------PG---------D-----------DLV-PQ----DL---------PDA------------------IRSG-------------------NV-------------------------------------------NDMD-LLVGSTMD**E**GTLFI-QYYLSSV---LDFT----DPESISKKAL-----KVYL-------SFLFRLIRQ----------------RST---QDIADQYVNSDP--NE------------SNA-------EFLRRA-------------------------GHSLGDFGFLCPMVDFAAD-------Y-AER------------------N--NSVYFYEFGYRPGY----SWN---------------E------------RWQG---VA--**H**FDEIPFIF--------GTNLD-TTEF-------------------HVTREERIFTLFMMHTWSHFAKTG

>CCE26

HKFFGIPFAEAPLGE--LRFR---------------HPVTVK-S----W-------SPKTVRANTKPFPCLQGPFYI-----NSN-----------------------LTIDT----ANSTEDCLYLNVWTPDDCVLN----------------------------GTCGAQKS--------------VMVFIYG------GTYTFGSSGWN-MYDGEQLALRGDV-VVVSFNYRVGPFGFLYSA---------TE---DAPGNAGLFDQLMALKWVQDNIRLFGG-----NPNDVTLF**G**Q**S**A**G**AISIAYH-MASP-L---TRG--LFHKVIMQSGSSYFRMTDPTRE--GPKKVEK-LANSMECLQ---DGELI-ETHAKEIVACLRKKD-GKELMETTFSAF------------GLNALTFFPI---------------HG---------D-----------EFL-PK----PA---------QEL------------------ITSG-------------------KL-------------------------------------------PKVP-VLIGNNKD**E**GSYFI-YYLFGRS---LSLA----DLDLVTKYEI-----DLYV-------TFGLQMLLQ----------------SNV---RNIRAQYFDGVK--EE------------ETM-------KALRQA-------------------------AIMVGDLAMVCPTKYFAEE-------A-AAQ------------------N--LTVHYYEFDFRSSF----GTW---------------P------------DWVG---TT--**H**GEEIPFVF--------GHPLS-GLEA-------------------NATAEDKEMSREMIKIWTDFAKTG

>CCE27

YSFLGVPFAEPPLGD--QRFN---------------KPVPYG-K----W--------DGIYVAETPSFPCLQDDQYY-----SER-----------------------MQVSG----ANSTEDCLYLNIWTPDLCR------------------------------QRACERKA--------------VVVLLHG------GGFFSGGNSYM-FNNGSFLSALGDV-VVVVPNYRLGIFGFLDLG---------TP---DAPGNQGLYDQLLALKWVQKNIESFGG-----DPSKVTLM**G**Q**S**A**G**AISAGLH-MISP-L---SRG--LFTRAIMQSGSPYARPSDGALQ--TISRLEA-LAVELGCSV---SQKD--TFKKEDIVQCFKWTE-AKKLLDASRRMGG-----------GMSGNNFFPT---------------WG---------N-----------EFL-PL----EP---------REA------------------MEQG-------------------DM-------------------------------------------EPVD-VLIGTNQN**E**GGAFI-NYFLHKV---LKQD----SMNAVSPEEV-----RFYL-------NVFMRFSLQ----------------SAA---RELSDFYFKGVQ----------------NTV-------QAFQSA-------------------------CLTFGDFLYQCPTNYFADI-------L-ERY------------------G--RKVYMYHFDHRPSF----SWY---------------E------------KWLG---AV--**H**FDEFVFVM--------GQLFH-GDLAF------------------SPTIDELRLSARMMKMWTHFIKFG

>CCE29

DTFLGIPFAEPPLGR--KRFQ---------------RPFRLR-P----W--------SGTLDALEYGHPCQQLNYTA-----GWE-----------------------WVFPK----KEQSEDCLTLNIWTPRRRLS-----------------------------KTVCPLKT--------------VLIFIYG------GGFNIGSSDWE-IYDGRTLSAYGDI-VVVTMNYRLGPLGFLNAN---------IR---DVPGNMGLYDQQMAIRWIYDNIKNFGG-----DPKSIVLM**G**E**S**A**G**SVAVGLH-LISP-M---SRY--MIRRAIMHSGSPLWDTPDNTID--GPKKANE-FAEIFGCTN---RSVTF-ESDKNTVLDCLSRID-AAELNAKSLEIL------------GKRVLTYHPR---------------WG---------D-----------QFL-PL----RP---------KDA------------------MRQG-------------------FF-------------------------------------------RDVE-VFMGVNRD**E**GSIFL-ANTLPEI---FMKG----PLPNITRDEA-----SLYM-------VFFFQYILR----------------SGT---RDIRDHYFSKFE--NN------------DFG-------GVRQAF-------------------------IDAIGDYLQICPTVYFGEY-------M-AEY------------------A--NNVFFYLFNHRPSN----SYW---------------D------------EWLG---VA--**H**FDEIQFVF--------GMPLR-YPD--------------------QFTKDEIELSKKMMKIWISFMKNG

>CCE28

DVYYAIPFALPPVGP--FRFR---------------KPVPVK-R----W--------KGILDATRPGPPCTQKDVQV-----NQF-----------------------YSRDS----VNSTEDCLHLNVFTAARYCFSQ--------------------------NLLHCGSKP--------------VIVFLHG------GGFQNGGNSDF-VLDSKHLAVMGDV-VVVIPNYRLNVFGFLNGNV--------TE---EVPGNMGLYDQILALEWIQKNIASFGG-----DPRRVTVFGQGAGAVSTGYH-LISP-M---SRG--LFKRAILQSGSPYWKLPDNTFT--GKMKVQQ-LALELGCDEYIVRGQVL--AYPREVMKCLRRIP-SHTLYEAMAKVF------------GSEGSTMIPS---------------YF---------N-----------ELL-PN----EP---------ITS------------------CEKG-------------------HY------------------------------------------ENNVD-VLVGTVTD**E**GTLPV-DQFFWKL---FALD----SFFSVPVSDI-----WFYA-------SLFFQSVLG----------------RSV---EPVRGLYADTNG--DNSS---------TSAK-------DAVEAV-------------------------AAAVGDFGVVCPSSYFADS-------Y-SKR------------------G--NRVWLYQFDHRPSY----SAS---------------P------------EWTG---VV--**H**GDDVGFAL--------GYPLW-QK---------------------SSTEEEKLLSRRVIRIYAHFAHTG

>CCE31

DTFLGIPYAQPPVGT--LRFK---------------APEAAY-A----W--------QNSINATEFKSACRQTASEW-----PHH-----------------------LTRHS----GSCSEDCLYLNIWTPSEKM---------------------------------GKPRA--------------VMVFFHG------GDFILGSPSWP-EYDGGLISAFGDV-VVASMSYRLGRLGFLDAK---------SH---SAPGNQGLLDQNMALRWIHDNIESFGG-----DRTQVTLFGNDAGAASIGLH-ILSP-M---SAG--LFKRAILQSGSAFWKLMYD----------------------------------------------------------------------------VYGPT---------------YD---------N-----------DFL-PL----NP---------HMS------------------ASMG-------------------IF-------------------------------------------NDVE-VMIGTANN**E**GAWEFMRSNQWRN--FSSVE----SFKAINLLKF----IKHFFE------EFQFKKLVGP---------------KAT---EGIYHYFASNLR--GN------------DIQ-------HNRQFV-------------------------FDFIGDYVHTCPSTHFAEL-------L-ADR------------------N--VSVYYYRFAHAPHTH--PETW---------------P------------LWVG---AT--**H**FDEVPYVF--------GAPLM-RSA--------------------EYSNAERDFSASVMDAWTSFAKTG

>CCE30

RVFLGIPYARPPEGK--LRFR---------------KPQPVN-F----T--------KGLRAAKGMAPSCLQLNEDL-----PPF---------------------PWLPEKN----TSFSEDCLYLNVWTPLTGR-------------------------------SKIERFP--------------VLIFLHG------GGFRSGSSSLE-VYDGGTLSAYGDL-VVVTVNYRLGSLGFLQLNS-------------KISGNMGLYDQVRALEWTQLHIGYFGG-----DPSQVTVMGQEAGAVSLGML-LLSP-L---CKG--LLKQAVLLSGAPNWLVDPMDTT-ESITRARK-LASEVGCVP---TSLHP-GKKLQQTGQCLMKVN-ASSLVEAEHRIL------------NGSYYGFLPR---------------QR---------D-----------MVV-PM----DP---------VMA------------------VAHG-------------------HV-------------------------------------------LPVD-VLVAFTED**E**GYMQT-YKLFPHL---FT------NESRLTLSEA-----RSIL-------AEVLDLFPD----------------DAT---DLLMDFYFGKLN--DS-----------DPPN-------DIRQAL-------------------------SQAFGDIFVTCPARIFAES-------F-GDR------------------P--MNAFLYEFKYKPLL----SMN---------------P------------AEIG---AT--NFDDIPYLF--------GEPLR-FPE--------------------RFASDDVSITKMMMNIVTSFVKSG

>CCE16

DAFLGIPYAKPPVGK--MRFR---------------HPVPMD-P----W--------EKPLNVTEPPATCVQVVDTYFDDFEGST-----------------------MWNAN----TNMSEDCLNMLVWVPRPR----------------------------------PTNAA--------------VLLWVYG------GSFYSGCATLD-VYDGKILASEENV-IVVSFNYRVGSLGFLYLD---------HA---DAPGNAGMMDQVMALRWVQDNIHLFGG-----NPNNVTLF**G**E**S**A**G**AVSVAYH-LLSP-L---SRD--LFSQAVLQSGGATVPWGYNERQ-TAITNGYK-LAEEVKCPT----------DDVEATIKCLRLQD-PDLLVKSEIFAT------------GVVDFSFIPV---------------VD---------G-----------AFL-TE----RP---------EDS------------------MSSG-------------------NF-------------------------------------------KKCK-ILLGSNRD**E**GTYFI-IYYLTQL---FKRD----ENVYLTREDF-----VDAV-------QALSPFTSS----------------VVN---EAIIFEYTDWLN--PD------------DPI-------KNRDAV-------------------------DKIVGDYYFTCPVIDMAHY-------Y-FIS------------------G--LDVYMYYYVYRSSQ----NKW---------------P------------EWMG---VI--**H**ADEIAYVF--------GEPLN-QTW--------------------SYRQDEQMFSRRIMRYWANFARMG

>Am_GB18414

DAWFGIPYAQKPLES--LRFR---------------HPRPAE-R----W--------SGILNATTLPNSCVQILDTVFGEFAGAT-----------------------MWNPN----TPLSEDCLYVNVVVPRPR----------------------------------PTNAA--------------VMVWIFG------GGFYSGSATLD-VYDHKTLVSEEKV-ILVSMQYRVASLGFLYFG---------TP---DVPGNAGLFDQVMALEWVRDNIAAFGG-----NPDNVTLF**G**E**S**A**G**AVSVSMH-LLSP-L---SRH--LFNQAIMQSGSPTAPWAIISRE-ESIVRGIR-LAEAVGCPH----D----RDNLQEVIDCLRVKD-PVELVKNEWGTL------------GICEFPFVPV---------------ID---------G-----------AFL-DE----TP---------QRS------------------LATS-------------------SF-------------------------------------------KKAN-IMMGSNTE**E**GFYFI-IYYLTEL---FHID---GSEVKVSREQF-----ISAV-------SELNPYVNQ----------------FGR---RAIIYEYTDWLR--PD------------DPH-------ANRDAL-------------------------DKIVGDYQFTCNVNEFAGR-------Y-TDT------------------G--NTVYMYYYKHRSMN----NPW---------------P------------RWTG---VM--**H**ADEISYVF--------GEPLD-PTK--------------------GYTPEEVNLSKKMMRYWANFAKTG

>Tu_19g00850

DAFLGIRYAKPPTGK--FRFR---------------HPKPID-S----W--------QGIFNATSFSGACYQVNDTFFGNFMGAT-----------------------EWNPN----VPLDEDCLSVNIWVPRPR----------------------------------PKSAA--------------VLLWIYG------GSFWSGSSSLD-FYDGSVLAGEESI-IFVSINYRVASLGFIFFD---------TS---DAPGNAGLFDQLMAMEWIRENIAAFGG-----NPANITIF**G**E**S**A**G**AVSAALH-LLSP-L---SRN--VFSQAILQSGSATCPWAISDRK-KAYQRSLA-LAQAVGCGS---TS----TRSVHAIIECMQSIP-ASELVAQEETTT------------GVVEFAFIPI---------------VD---------G-----------SFL-DE----DP---------EVS------------------LRTK-------------------NF-------------------------------------------KHTP-ILTGSNRD**E**GTYFL-VYHSPHI---FNLS----EGIYISRSEF-----QSLI-------RIIYPHLSP----------------LAQ---EAVIQEYTHWIN--PD------------DQI-------ENREAT-------------------------DKFVGDYHFTCPVNEMSYR-------Y-ALY------------------G--NDVWTYHFTHRSSK----SFW---------------P------------SWMG---VI--**H**GEEIKFVL--------GEPLD-PVH--------------------GYTPAEVQLSKRIMRYWANFARTG

>Dm_CG17907

HVYTGIPYAKPPVED--LRFR---------------KPVPAE-P----W--------HGVLDATRLSATCVQERYEYFPGFSGEE-----------------------IWNPN----TNVSEDCLYINVWAPAKARLRH-GRGANGGEHPNGKQADTDHLIHNGNPQNTTNGLP--------------ILIWIYG------GGFMTGSATLD-IYNADIMAAVGNV-IVASFQYRVGAFGFLHLAPEMPSE--FAE---EAPGNVGLWDQALAIRWLKDNAHAFGG-----NPEWMTLF**G**E**S**A**G**SSSVNAQ-LMSP-V---TRG--LVKRGMMQSGTMNAPWSHMTSE-KAVEIGKA-LINDCNCNA-----SML-KTNPAHVMSCMRSVD-AKTISVQQWNSYS-----------GILSFPSAPT---------------ID---------G-----------AFL-PA----DP---------MTL------------------MKTA-------------------DL-------------------------------------------KDYD-ILMGNVRD**E**GTYFL-LYDFIDY---FDKD----DATALPRDKY-----LEIM-------NNIFGKATQ----------------AER---EAIIFQYTSWEG---------------NPGY-------QNQQQI-------------------------GRAVGDHFFTCPTNEYAQA-------L-AER------------------G--ASVHYYYFTHRTST----SLW---------------G------------EWMG---VL--**H**GDEIEYFF--------GQPLN-NSL--------------------QYRPVERELGKRMLSAVIEFAKTG

>Am_GB14873

HVFYGIPFAKPPIGP--LRFR---------------KPLPIE-P----W--------HGVLNATVLPNSCYQERYEYFPGFPGEE-----------------------MWNPN----TNISEDCLYLNIWVPQKYRLRHKGDGSPGG-----------------NGGPRNGLLP--------------LLVWIYG------GGFMSGTATLD-VYNADIMAATSNV-IIASMQYRVGAFGFLYLNKHFTN----SE---EAPGNMGLWDQALALRWLRDNAEAFGG-----DPELITIF**G**E**S**A**G**GSSVSLH-LISP-V---TRG--LVRRGILQSGTLNAPWSYMSGE-KANEVATI-LVDDCGCNS-----TML-NENPARVMACMRSVD-AKTISVQQWNSYW-----------GILGFPSAPT---------------ID---------G-----------IFL-PK----HP---------LDL------------------LREA-------------------DF-------------------------------------------KDTE-ILIGNNEN**E**GTYFI-LYDFNDI---FEKD----QASFLERERF-----LGII-------NNIFKNMSQ----------------IER---EAITFQYTDWEE--VY------------NGY-------IYQKMV-------------------------ADVVGDYFFICPSIHFAQL-------F-ADR------------------G--MKVYYYFFTQRTST----NLW---------------G------------EWMG---VL--**H**GDEVEYVF--------GHPLN-KSL--------------------KYSDKERDLSLRMILYFSEFAYLG

>CCE17

EAYLGIPYAKPPLGD--LRFR---------------KTVPVE-A----W--------AELKATKDFSKPCVQATYIH-----GQN-----------------------VSVDY----SDSSEDCLTINVWKPAGG----------------------------------SAKKA--------------VIVYIYG------GAFQFGDSSLF-YNDGLPFSALNDV-LFVSFNYRVGVFGFLSTH---------HS---DAPGNEGFYDQLMALKWIKQNVEVFGG-----DPEAITLF**G**Q**S**S**G**AFSVGLH-MYSP-L---SKG--LFKRVVFESGTALSLITFQMKN--EISRFHT-LIGLTNCSY----GIYS-ESKYNETMECIRKAE-AKSIIEGVSHL-------------GHLADMFLPI---------------YN---------D-----------DFI-PV----HP---------LMP------------------EEVK-------------------AL-------------------------------------------NGDE-VFMGTVTD**E**GTMFI-NYPLQNL---LAQG-------DIAKTQY-----KIVP-------SSIYPRLFDA-------------PWGAI---YHNFDQYFENSI--DS-----------YPEE-------TTYNLI-------------------------GQTYGDAVFTCPTNLFGQF-------L-AKR------------------K--RSVYRYQYTHKSSL----KLW---------------P------------DWMG---VV--**H**TTDLPFMI--------GSMLT-HEANLAEPRYNNTP--EWFRKH-KFTDEEIKFSKQLVKTLGDFVKTG

>CCE18

EAFLGIPYAQPPVNE--LRFA---------------KPVPVK-A----W--------NGILEATRKRFGCPQANQFL-----SPH-----------------------IQLLY---ENHTSEDCLTLNIWRPKLS--------------------------------EGSAPKP--------------VLVFIYG------GAFQWGSADVF-LYDGAANSALNHH-IFVSFNYRVGPFGFLGHPD--------LP---AISGSIGFWDQNTALRWVKDNIQSFGG-----DPDQITVC**G**H**S**A**G**AISAAVH-AISP-H---AKG--LFRRAILQSGTTLSIGTLSRIP---RDTLRK-IAEQLGCEG-----------NPSELVDCLRTFE-ASELVAGIEALG--------------LEGLILPP---------------YE---------S-----------DYV-SL----SP-------------QF---------------LENA-------------------TF-------------------------------------------NVDE-IIIGTNEH**E**ARHFA-RRIVDSN---RSLK--AIFAEIGGMAPF----------------AGLLRNIFEI-------------SISEA---RSIVSEYFPVHE--EL------------SES-------EVIDLA-------------------------SQAATDLFFLCPAHFFSQV-------A-ASK------------------N--VAVFRYLFRHRPSF----TLY---------------E------------EIPG---VL--**H**GDELLFTM--------GNLPV-IQELLREDIVARAD--EYVSKL-NHSTEEQDLFENIQNTWSSFVRNG

>CCE19

EVFYGVRYAEPPVGD--LRFR---------------HPVPRA------Y-------SGASLDATKPGHACIQGNTFY----IRSN-----------------------VSFGF----AETSEDCLHVNIYRPKGA-----------------------------------QNAP--------------MAVYLHG------GTFVAGFNKLF-LYDAEELAARENI-IIAVVNYRLSVLGFLYLN---------HT---NAPGNQGLFDTLLAAKFVQQNARALGG-----DPDRITLW**G**Q**S**A**G**GMAAGFL-LASP-L---AKG--IFKRAILQSGVTAAATPALRLN--NVNNAVS-AASVLDCVD---SNRTV-DEQLGDMAQCMKKID-AQRLFDAVNVDLG-----------ERYTITFQPM---------------YGI--------D-----------DML-PV----FP---------FPA------------------NYSE----------------VPLND-------------------------------------------DVKE-VLTSTVSD**E**GAMFI-WGVLERF----------QYADSSSVEDF-----ISLS-------QVVLKVVLEM-------------PAEYV---KNGIPRYISPSV---------------TDQA-------EMRKQV-------------------------SDLFRDVLFECPVDMYSDF-------L-ATR------------------G--VKVYRYLWNHKPST----SYW---------------P------------EWAG---AT--**H**CDDIPFVM--------GSQFD-IGNK-AEKSNQASAGLVHFLKT-PITEAEKQLIRDSLKMIGDFVKTG

>CCE20

ETFFGVRYAQPPVGD--LRFK---------------HPMPIA------Y--------IGERNATVMGPACIQRDEFF----IRRN-----------------------MSYRF----HPMSEDCLHLNIYKPKNA-----------------------------------RGLP--------------IALHFFG------GSFYGGYNGHY-IHDPEQLVAQEKV-IVVTANYRVGPLGFLYLN---------SS---HAPGNAGIYDMLLAARFIKQNAEALGG-----DPEKLTLW**G**Q**S**A**G**AIAVSML-MSSP-L---AEG--LFNRAILQSGSAPSAALSLHMN--SASNGIY-AASVLGCRS---SADEVDERDIDEIATCMKLID-GKKFFDTLQSAAG-----------DQFLVNFQAM---------------AGI--------D-----------DLI-PE----YP---------FRG------------------AQSK------------------LNA-------------------------------------------GIEE-VLIGTIQN**E**GGMFA-NSAMERA-----------GYDTPSAENF-----LALA-------WFMLKSVLSV-------------PAHFV---RRAVFTYIRDDS---------------EDVE-------ELQDSM-------------------------SALLGDVLFECPADLYSTF-------M-DNQ------------------G--VKVYRYMWAHRPST----SAW---------------P------------EYIG---PT--**H**YDDIGFTM--------GSQLH-IKDR-AETSGQASHGFLDCFSR-GITEAEESLLRESLSMIGNFLRTG

>CCE21

EVYHSVPFARPPVGD--LRFE---------------RPVPYE-D----P--------EAVVDATKEHPDCPQHLVAL-----SEK-----------------------TFINF----TRNSEDCLYLNIFKPVTN----------------------------------ETDLP--------------IVYYIYG------GSFLHGSANFF-LTDGDILATETNV-IYVIVNYRLGVFGFLTSG---------GS---MAPGNQGLWDILEGLRWTKKNARAIGG-----NPENITIW**G**H**S**A**G**AIAAGIF-MTSP-I---TEN--LFHHAILQSGATKTGHVLFQQN--SPEVTAR-TLVRLGCLQ---DADQY-ERSRDKAIRCAKKLD-VETILSELAPKGF-----------DLDIISFQPV---------------IG---------D-----------DLI-PV----DPF-----------------------------VKRQ-------------------KL------------------------------------------HSAKS-LLVTSCAT**E**GVVFL-EILRVRG-----------ADLVEGVGDL-----RFMM-------ALGTKQLLQI-------------PVSAA---SKIAYSYFDDQS--EP------------DLK-------SAETIL-------------------------QRYISDMLFICPIEFFTDA-------A-EAN------------------G--IPVYRASFEARPSW----SPW---------------P------------SYSV---AA--**H**GDDLPILS--------GQALG-YAKNVKKSYVVPDVGDL------NMTAAE------------------

>CCE24

SAFMGIPYAQPPLGK--LRFQ---------------RPVPIS-R----K--------GGVFDARQARPGCLQLDLKY-----SEV-----------------------VDIDN----SDTVEDCLHLNVFAPRRSDS--------------------AEDYEDEA-PKQDVKLP--------------VFVFLYG------GVYVWGENQLA-LYDGVEFAAEADA-IYVVVNYRLNIFGFLKSP---------DD---RIPGNVGLWDQLEALKWVRENIASFGG-----DPEMVTLG**G**Q**S**A**G**AFSAAIH-TYSP-L---SRG--LFKRAFLMSGSSFSLKFLQRTS--TDDLFGT-VANTLSCLD------------GVDPVDCLTTRNLTRNSLKSIENEL------------RTRFMNLVPSS--------------VD---------------------QLM--D----HP---------VDE------------------LHRN-------------------KY-------------------------------------------HIKE-VMLGITLR**E**GDFFI-NNFGRRL---PFFS----DLLGQTPQAA--------I-------RLAMKVLFGI-------------GIRES---AEIYNAYFADSATGEF------------SSH-------ELVRKV-------------------------SDIVGHCVFDCPTEIFARH-------A-AAS------------------G--VKVRHYVYAHQPSV----KIN----------------------------LNTGN-EPT--**H**IDDIPFML--------GSINS-VAENSRTLRMK-LPKFFLDAQK-KFSPEEHHFSRVVIRQIANFMRSG

>CCE25

NVFIGVPFAKPPLGD--LRFK---------------RPEPLE-P----S--------DKEIDALEKKPMCPQTPMKL-----------------------------PGIVLEN----TFNDEDCLYMNIFASAEST---------------------------------EKLKP--------------VMVYLYG------GFFQWGDNNLG-LYDGVEFAAETES-VLAIPSYRVNMFGFMNST---------TE---TAPGNAGLFDQLMAMKFVKRNAKAFGG-----DPDLITLA**G**Q**S**A**G**AISTSVH-TSSP-A---SKG--LFRRAMMLSGAASTLAFFSEAN--GQTLLFT-ISNFLDCFD---GNLTI-GEQYDQMAKCLKTLD-KETLLGQMKKLS------------GADSLAFGPG---------------FD---------G-----------EII-AGSLK-RP---------SEL-----------------------------------------QY-------------------------------------------NVED-IMLGSTAA**D**GELIV-KQVMGLD---ASIE----DLARNSGADI--------------ALRLALRHLFGI-------------NNKDA---YSIYHQYVTDDE--HN-----------AGYD-------AVFQKA-------------------------PRLVTDVSFDCPIKFYMNS-------V-EKK-----------------NGKKTRIYRYALDGPRAK----AFS----------------------------NSMGDGPAT--**H**TDDVMFFL--------GIALN-KKVE--RPWNPTNPEALQQTLD-GYTDVDRQFAKQLLASIRDFMVEG

>CCE22

NSYLGVPYAEPPTGQ--RRFM---------------KPIPLL-P----T--------PGVFNATRIPPLCMRPSRME----------------------------------------LKMSEDCLYLNLWIPSKPDGNP-----------------------EQMKAARGMNVP--------------IVVYIPS--F----------DQII-IPDGAEFAARNDV-AFISLNYRVGALGFLFSG---------SP---EAPGNAGLYDILEALRWVKLNSRLFGG-----NPGDITLWAKGEGAAVAAVL-MTSP-M---TKD--LFQKIIFESGSVHTSRNSFKTN--SEAVTSR-LLVNAGCFN---YSKTW-ESQRDEAIRCLKVVH-PEVIIGSQ----------------NFEPDAFQPV---------------IG---------D-----------SLI-PD----EP---------FQG------------------DRQH-------------------FT-------------------------------------------NPYQ-LFILMKDD**E**SDPTL-TDLLRSQ-----------PGSVDTTSDC-----KHVL-------RTFLRTSLNI-------------PLNFV---DKLLLRYKRDIQ--------------------------DARELC-------------------------RQVFTDAYFMCPVKIFADV-------L-SGM---------------------RDTSSVLFAKRSSL---VDEF----------------------------ELSG------------------------------------------------------------DVDEKLLDILGRFVKTG

>CCE23

AAFLGVPYAQPPVGA--LRFA---------------APRPPL------F-------SDGIYNATEKKFSCPGPEISI-----GKW-----------------------VVADF----GEQDEDCLYLNIWAPANA-----------------------------------SNLP--------------VVLYIHG------GIFVAGSGGWF-PFDGTRLAPRADI-VYITMNMRLGVLGFINLPV-------------LSDTNVGLLDIVRAVHWVHENAEAFGG-----NPEQLTLW**G**Q**S**A**G**AISIDFL-LKSD-A---ISG--LVRRAILEGGSAGLAKFLLEFD--GEYESRQ-LAYTLGCSS---AEPDG-EAHDRELLSCLREVP-WQDIIKTRSEGG------------TIDKVSIQPF---------------ID---------G-----------KLL-KKDVVASP-----------------------------------------------------HI------------------------------------------DPSVEELFVVSNEL**E**AFLIL-EGLVKKL---YETN----DITSIDWGTIL---------------KISFKVVLRL-------------PYEKS---AEIIRRYIPDDVI-DESR-----LSKELREI-------EIQRGI-------------------------ASMFTDSFFYCPAEFRSAT-------A-GDR------------------G--VRVSRGEFHFRPDF----SFV---------------P------------KWAS---MS--**H**AEEIALMHGNLDLYKPGKGLS---------------GAMMNSFV-NVSRSDYDFSDVIVKWIGDFFH-G

>CCE34

RQYFGIPYARPPVKK--LRFQ---------------KPRPLP------WR------KSRVILATRMGPSCPQTPYYF-----DDT-----------------------RNNRH----MRFSEDCLYLNVWTAQLA-----------------------------------PLRP--------------VVVFLHG------GFFTHGGSESP-NLDMSHLAARG-V-VAVSFNYRLNAMGFLYAG---------VP---QASGNMGLYDQQLALQWIRKHIHYFGG-----DPARMTVMGHGAGATAAAYH-LLNP-T---SRK--IFKRMILLSGNPFSIMNLNMPN-VASTRAST-IAKRLGCSR---DGNM--KSESFEIFHCIASKD-ANEIAKAAELEF------------TRGELSLMPL---------------FT---------SE----------DFL-KK----SP---------EQL------------------VYES------------------SKM-------------------------------------------DDVE-ILMGHANN**E**YADSL-----------------YYTGYFDAADKILPGDIKYLI-------GMFYGMFFR----------------ANA---LPIIRYYFRNDR--TA------------NAS-------SLLRTG-------------------------GRAISDGLLVCPGLEYADE-------M-SRL------------------G--AKVYYYSFDHSSAFS---------------------K------------AQFG---AT--SSEEVLYFL--------GSILG-----------------PLGTQL-GASRKDRQFAEDMLDVVTTFVYYG

>Dm_CG1121

YSFEGIPFAKPPVGE--LRFK---------------APVEPE-H----W--------SDVKRCTHVRAKPCQVNIVL----------------------------------KQ----VQGSEDCLYLNVYTRELHP---------------------------------HRPLP--------------VLVWIYG------GGFQMGEASRD-LY-SPDYIMMEHV-VLVVISYRLGALGFLSLA---------DEE-LDVPGNAGLKDQVMALRWVKRNCQFFGG-----DPDNITVF**G**E**S**A**G**GASTHYM-MLTD-Q---AKG--LFHKTIIMSGSALAPWAQTPTH---INWPYR-LAQATGYTG---------DANDRDIFAHLKKCK-ASSMLKVAEDIITMEERH-----QRLTMFSFGPT---------------IEPYLTP----H-----------CVI-PK----SP---------LEM------------------MRDC-----------------WG---------------------------------------------NSIP-MVIGGNSF**E**GLLMF-PEVNKWPELLCQLGD-----------------CENLA-------PQDAH--VD-----------EQQRKAFG---KKVRELYFGDRT----------------PGR-------KTILEY-------------------------SDLFSYKYFWHGIHRTLLS-------R-AHH------------------APLAPTFLYRFDFDSKH---FNIMRIITCGR---------------------KVRG---TC--**H**ADDLSYLF--------YNAAA-KKL--------------------KRRTAEFKTIKRLVSMVVHFAISG

>Dm_CG2505

FAFEGIPYAKPPVGD--LRFR---------------APQPPE-P----W--------QGVLNCTTNRSKPMQRNMLL----------------------------------GI----VEGSEDCLHLNVYVKALKS---------------------------------EKPLP--------------VIVWIYG------GGFQKGEASRD-IY-SPDYFMKKPV-VFVAINYRLAALGFLSLK---------DPK-LDVPGNAGLKDQVMALRWISQNIAHFNG-----DPNNITLM**G**E**S**A**G**SASVHVM-MTTE-Q---TRG--LFHKAIMQSGCALSEWVESPDN----NWAFR-LAQNLGYKG---------DEKDADVLSFLSKVC-ARQIAAIDQDVINLDEVR------SFLLFAFGPV---------------IEPYETD----H-----------CVV-PK----RH---------KDL------------------LSEA-----------------WG---------------------------------------------NDIP-VIVGGNSF**E**GLFSY-QLVRKDP---WALKN-----------------FHNIL-------PREVRETSS-----------LEGQDLLV---RRLKQLYFNNEM----------------QES-------MEMFEA-------------------------LNIFSHRQIWHDTHRFILA-------R-QSY------------------APKTPTYLYRFDFDSPH---FNQFRRLVCGD---------------------RIRG---VA--**H**ADELSYLF--------YNIIA-SKL--------------------DKSSMEYKTIERMVGMWTSFASSG

>Am_GB11064

YSFKGIPYAKPNVGP--DKFQ---------------ISEPAE-P----W-------EDQVYDATMHRSACAF----------------------------------YCKVKKG----IIGEEDCLYLNVYTPVLDK---------------------------------EARKA--------------VMVWIYP------GGWNGGLGDDI-LF-GPDFLVEKDV-VLVTFNFRNGALGFLNTE---------DK---SAPGNAGMKDQVLALKWVKDNIHYFGG-----CPNRVTIF**G**D**S**S**G**GASVQYH-MLSP-M---SEG--LFNAAIQQSGTILNPWAITYNP---REQAFM-LGEALGIKT----------TDSEELVRKLSEFH-VEDIIAASSEIMKKQNVL------SGHTNAFVPS---------------IEVDMGQ----D-----------VFL-PT----DP---------WTL------------------LKSG-------------------RI-------------------------------------------ADVP-VMSGITAD**E**CAFMA-QNMIDKI-----------DVLNTEPEQFL---------------PDDLNYTDSNT------------KKESG---QSLKKFYFGEKQ----------------VSK-------DNLNEY-------------------------IRMLSDIFFDAGELLSLDI-------M-KNRI-----------------S--SPIYQYLFSYEAPT----GFMKSLHG-----------------------VSDG---VA--**H**ADDVGYLF--------YSNIF-KNLP-------------------EPDSSAEKVINIMTKMWTNFAKDG

>Am_GB16342

SSFKGIPYASPPIGN--RRFR---------------PPVPPQ-P----W--------NETLDAIEEANECPQEMSNV----------------------------------------YSGNEDCLYLSVFTPQTKFND----------------------------KELKTLKP--------------VMVWIYG------GSFLRGSNNAS-LY-GPDFFMEQDV-VLVTFNYRLGALGFLYLK---------HE---NAAGNAAMRDQLMVLEWVRDNIAAFGG-----DPNRVTLF**G**E**S**A**G**GASVNYH-VLSE-K---SRG--LFHQAIEQSGTSATYLYKTQKA--AFQTACK-LASELGFES----------DDPNELLKFFLEAD-AKDLVATTNRAFPLG---------TDFSVPFAPI---------------KENPDLVD-PKD-----------MFL-SE----CP---------ITL------------------AANQ-------------------KF-------------------------------------------NKMP-VMLGFTHD**E**VLDFS-GELYQII--------------------------------------------------------------NTT---ADILNELFNLKL--DL-----------QGPY-------EEVKEL-------------------------SVVLSDFIMKGPIDFAQRL-------L-VDG------------------NDDYPVYYYQLSYVSNY----ALH----AQDGI------P-------------EPG---IA--**H**FDDIGLLF--------NVESL-NAPT-------------------DPRHPFNQFRQKLVTLWANFAKYG

>Dm_CG8425

EAFMGIPYALPPIGD--LRFS---------------NPKVMP-K----L--------LGMYDASAPKMDCIQKNYLL--------------------------------PTPV----VYGDEDCLYLNVYRPEIR----------------------------------KSALP--------------VMVYIHG------GGFFGGSAGPG-VTGPEYFMDSGEV-ILVTMAYRLGPFGFLSTQ---------DA---VMSGNFGLKDQNLALRWVQRNIRFFGG-----DPQRVTIF**G**Q**S**A**G**GVAAHMH-LLSP-R---SHG--LFHRVISMSGTANVPFAIAEQP---LEQARL-LAEFADVPDAR-------NLSTVKLTKALRRIN-ATKLLNAGDGLKYWD---------VDHMTNFRPV---------------VEEGLEV----D-----------AFL-NA----HP---------MDM------------------LAQG-------------------MP-------------------------------------------TSIP-LLLGTVPG**E**GAVRV-VNILGNET--------LRQSFNLRFDEL----LQELLE---------FPASFS-----------QDRREKMM---DLLVEVYFQGQH---------------EVNE-------LTVQGF-------------------------MNLISDRGFKQPLYNTIHK------NV-CHT------------------P--NPVYLYSFNYQGPL----SYASAYTSANV-------T------------GKYG---VV--**H**CDDLLYLF--------RSPLL-FPDF-------------------QRNSTEAKVIHSFVDYFVHFAKFG

>Am_GB10820

EAYEGIPYAQSPVGK--FRFQ---------------PPRPIK-K----W--------SKDLSATKKSSVCMQYLMTF------------------------------TTHGNR----VKGSEDCLYINIYVPVRN--------------------------------NRKPLLP--------------VMFWIHG------GAFQFASGNEA----NETLFMDRNI-VFVAINYRLGPFGFLSTG---------DI---VVPGNMGLKDQSMALRWVFNNIKSFGG-----NPNKITIF**G**M**S**A**G**GASVHYH-YLSP-M---SAG--LFKRGISISGVAFCPWAQTKHA---PEKAKK-LGALMKCRT----------DNTKKMIDCLQSRP-ARIIAQAVGDFMFWL---------YNPFTPFGPV---------------VETYGS-----N-----------PFI-SN----SP---------INI------------------INNG-------------------QV-------------------------------------------YDVP-WISGVVSK**E**GLYTA-AEFVDNAK--------LLWHLNDHWDEI----APYLLD---------FNYTIP-----------LDQHRQVA---KKIKNYYLRSGPI-NY----------------------DKVESI-------------------------IQMMSDRLFNIDFEKAVRL-------Q-ARI------------------N-KSPVWTYYYSYRAEH----SVSEILSG--------------------------------------------------------------------------------GSTTDYVNFTNIKMEVNNNFANKS

>Am_GB15327

EAYEGIPYALPPVGK--FRFK---------------APQKIP-A----W--------IGELSATKFGFPCLQYTQLP------------------------------VNPRDK----IEGAEDCLYLNVYVPADR--------------------------------TPSQSLP--------------VIFWIHG------GAFQFGSGIPM----GAKYLMDSDV-IFVTINYRLGILGFLSTE---------DE---VVPGNMGLKDQSMALRWVSENIEWFGG-----NPKRITLI**G**L**S**A**G**GASVHYH-YLSP-L---SAG--LFQGGISISGTALNCWTQTENS---LEKAKQ-VGAFMGCPT----------RNVKEMIRCLRYRP-ARAIVETLANFMRFY---------YNPFTPFGPV---------------TEKVNNDS---N---------SLPFI-DR----TP---------IEI------------------INSG-------------------DV-------------------------------------------QDVP-WVTGVTSE**E**GLYPV-AEFIAKPE--------ALKLLDENWDLI----APYFLD---------YNYTIP-----------KEKHVEVA---RLIRNYYFESNKI-DE----------------------TTLKHL-------------------------IDVASDRFFITDGEKAARM-------Q-AKV------------------N-RQPVWFYYYTYKGAH----SISEIMSGT---------S------------NKYG---VC--**H**ADDAYMVV--------DTPFL-AS---------------------TTTTNDIKMQKVLIDFWVSFVNNG

>Dm_CG17148

YSYESIPNAEHPTGA--LRFE---------------APQPYSHH----W--------TDVFNATQSPVECMQWNQFI-------------------------------NENNK----LMGDEDCLTVSIYKPKKPN---------------------------------RSSFP--------------VVVLLHG------GAFMFGSGSI---YGHDSIMREGTL-LVVKISFGLGPLGFASTG---------DR---HLPGNYGLKDQRLALQWIKKNIAHFGG-----MPDNIVLI**G**H**S**A**G**GASAHLQ-LLHE-D---FKH--LAKGAISVSGNALDPWVIQQGG---RRRAFE-LGRIVGCGH---------TNVSAELKDCLKSKP-ASDIVSAVRSFLVFS---------YVPFSAFGPV---------------VEPSDAP----D-----------AFL-TE----DP---------RAV------------------IKSG-------------------KF-------------------------------------------AQVP-WAVTYTT**E**DGGYNA-AQLLERN---KLTGESWIDLLNDRWFDW----APYLL---------FYRDAKKT----------IKDMDDLS---FDLRQQYLADRRF-SV----------------------ESYWNV-------------------------QRMFTDVLFKNSVPSAIDL-------H-RKY------------------G-KSPVYSFVYDNPTDS----GVGQLLSNRT--------D------------VHFG---TV--**H**GDDFFLIF--------NTAAY-RT---------------------GIRPDEEVISKKFIGMLEDFALND

>Dm_CG6917

YSYESIPYAEPPTGD--LRFE---------------APEPYKQK----W--------SDIFDATKTPVACLQWDQFT-------------------------------PGANK----LVGEEDCLTVSVYKPKNSK---------------------------------RNSFP--------------VVAHIHG------GAFMFGAAWQ---NGHENVMREGKF-ILVKISYRLGPLGFVSTG---------DR---DLPGNYGLKDQRLALKWIKQNIASFGG-----EPQNVLLV**G**H**S**A**G**GASVHLQ-MLRE-D---FGQ--LARAAFSFSGNALDPWVIQKGA---RGRAFE-LGRNVGCES---------AEDSTSLKKCLKSKP-ASELVTAVRKFLIFS---------YVPFAPFSPV---------------LEPSDAP----D-----------AII-TQ----DP---------RDV------------------IKSG-------------------KF-------------------------------------------GQVP-WAVSYVT**E**DGGYNA-ALLLKER---K--SGIVIDDLNERWLEL----APYLL---------FYRDT-KT----------KKDMDDYS---RKIKQEYIGNQRF-DI----------------------ESYSEL-------------------------QRLFTDILFKNSTQESLDL-------H-RKY------------------G-KSPAYAYVYDNPAEK----GIAQVLANRT--------D------------YDFG---TV--**H**GDDYFLIF--------ENFVR-DV---------------------EMRPDEQIISRNFINMLADFASSD

>CCE36

VAFLGVPFAAPPLSN--LRFK---------------RPQAHL-G----W--------NGVWDGTEFRDVCPQLDYRG---------------------------------------RPVGSEDCLFLNVFTPGIKK---------------------------------GTTFP--------------VLVFLHG------GNFDSGSGNYY----GPQALVDQNL-VVVTLNYRLGILGFASTE---------DE---ALPGNLGLRDQLLALQWVRDNIAVFGG-----QPDRVTLMGQG**S**GAICVMLH-AVSP-Q---SKG--LFHRLIVQSGTPLAESALHVNA---RETLQNAAGSLQSCEI----------LSSHDILACLTKTP-IRDLLRLQQETKIF----------ADYPVKYTPR---------------IDSDLS-----D-----------ALV-PS----SF---------SQL------------------LSSG-------------------VP-------------------------------------------VKVP-TLIGFNMH**E**TAFLY-PELLALL-----------RTELGSDQDYV---RNILL-------PKYFRTALGV----------REPTRDQL---DEIYESYFSGIN--YK------------DHD-------GIIQAF-------------------------VNMSTASLYSVPILETAIM-------L-ARS------------------S--PQTFLYSFSYRGDI----SMLDSKLG----------P----------RRVNLG---PS--**H**GDELLYLFQVL-----GNGLRP-----------------------STNLVDGMISRRMIRMWSDFAHGG

>Tu_04g06380

FGFLGIPYAKPPIGP--LRFK---------------QPVPHP-G----W--------SGLADAWTYKASCPQLDTRG---------------------------------------VDTGNEDCLYLNVYTPSVERT-----------------------------PFNNFLYP--------------VMVFIGA------GTFETGDSSLY----GPQKIMDKGI-VLVTFNHRIGLLGFLSSD---------DE---SASGNWGLYDQLLVLQWIKSNIESFSG-----DPSSVTIFGQGAGAASVFFH-LISP-L---SKG--LFHKAILESGSGLCDWALQSDP---WDYAVQ-IGQRLRCPI----------TPRDTLIECLRVVP-TLDLLRAQSNGKIL----------GEYPIKTVPV---------------VEKGTT-----N-----------RFL-PQ----DP---------WQL------------------LTSG-------------------IV-------------------------------------------NRVP-VLIGYNTQ**E**TLFFY-PEIEQAF-----------REESGRAHEKL---TSDFLDCCTPFK--------------------GRSKERVV---PLILYEYFNKMD--PF------------NST-------QVASRF-------------------------IDLSTDALFISCIDETVRI-------L-WEL------------------N--LPVYLYIFDYRGQN----SMVERIISKALI------P------------VDTG---PS--**H**GDELFYIF--------DMKLG-TSR--------------------NQPFRDKKVSQRITSLWTDFAKFG

>CCE39

VAYLGVPYAEPPLGL--FRFRRGKSRLELMPGLFFKEPQPLT------F--------KGNVEAKKFRPACLQHTNYT------------------------------GSSKGI----EHVSEDCLYLNVYTPNVSDE-----------------------------VNRGQRYA--------------VMMYIHD------GEYSHGSGNV---FPGHMLAATQEV-IVVTFNYRLGALGFLSTG---------DN---SSAGNFGLLDQRAAINWVYHNVERFSG-----DPERITIFGPGAGASAAGIH-MMQQ-IY--GEN-LHIKRVIAMSGSAVAEWASIDDAIFVRNISRL-YGEQIGCWA----------TDSWQLVECLKRKS-NNSVEFTLTTVTP-----------LRGWLPWGPV---------------LDRNTRYK---S---------------PG----MPY--------SAL-EY---------------LENKI--------------PRPERV-------------------------------------------QQFS-YMTGVSVN**D**ASFIV-ENDEELK---NIGWQLNKDAFDQKIRAY----------------AKTYNYTIN--------------EDAII---QAIRFMYTPWTD--SE------------NSS-------LLLEEY-------------------------VNMFTDALYVAPMDRMVKL-------L-LEI------------------E--VPVYMYVMNYSLTS----SYF--------------LPQ---------RRTWNR---VP--**H**DTESILVS--------GAPFM-DPKFYPSEY--------DFNKV-YWSEGDRNMSQLLMEAWANFAKEG

>Tu_30g01560

AVFLGIPYARPPLTEHGLRFK---------------SPQPPS------Y--------KGSWSADRFRPSCPQPSQYT------------------------------GADKMI----YEVNEDCLYLNIYTPKASE-------------------------------NKEKPSP--------------VMIYIHD------GNFIHGSGNR---FPGHMLAASQDV-VVVTFNYRLGILGFFATA---------DE---SSPGNYGILDQIQLIHWVRENIRKFGG-----DPEMITLFGPGAGAASAGIL-AISP-L---SRK--YIKRVIAQSGSAVAPWASINEPIMIRNMSIV-VGAAYGCST----------PLTRNLVECLKSRS-SSDIPIAAVANQV-------------GWLPFAPV---------------PDMTTRPL--GT-----------EVL-PD----TP---------EVI------------------LQKG---------------FPVDDI-------------------------------------------HLDG-YLTGVTRD**E**GSAMV-YSDEEIK---LNDYQVTLETLEEKIEKY----------------LKIYNATLN--------------PEAFK---SALRFMYTPPVD--PT------------NGT-------QIRQGL-------------------------IDMYTDSWNVAGVDKMMKL-------M-VKN------------------K--IKTYMYVLNYTIES----LHW---------------P------------RWMG---VP--**H**DTEYFLIS--------GAPFM-DDQFYPKMY--------HLDRV-KWKEDDRNMSTFFMKAWADFARHG

>Dm_CG3903

SVFLGIPYALPPTFE--GRFK---------------PPRVHR-G----W---------QLLQAVDFGPACPQPVRYT------------------------------GATKGI----MDMDEDCLYLNVYSPKTGA-------------------------------GVAQKYP--------------VMVYIHG------GEFIRGASNL---FQGHILASFYDV-VVVTLNYRLGALGFLSTG---------DE---NSPGNYGILDQAMALRWVYDNIEFFNG-----DRNSITLFGPGAGGASAGLL-MVAP-Q---TRN--IVRRVIAQSGSALADWALIQDKYRAQNTSRV-LGQLLGCSI----------ESSWKLVNCLRTGRSFYELGNAEFSPQV-------------GSFPWGPV---------------LDHNFTLP--GDDWYEGWREKDWRFL-TQ----TP---------ETL------------------IRAG-------------------KF------------------------------------------NRNIQ-YMTGVTTQ**E**AAFFV-AQNESLS----PYYELDGRFFDQKIREH----------------VFRYNYTLN--------------PNGVY---EAIKYIYTFWPD--PN------------NNT-------IIRDQY-------------------------INMLSDLYYRAPVDQMVKL-------M-LEQ------------------K--VPVYMYVLNTTVEA----LNL---------------P------------QWRK---YP--**H**DIERYFLT--------GAPFM-DTEFFPKKE--------HLQRN-MWTDNDRNMSHFFMQTYTNFARYG

>Am_GB12309

NVFLGIPYAMPPTKE--GRFK---------------PPRPHK-G----W---------QLLQAVDWGPACPQPSAYT------------------------------GATKGI----RDVDEDCLYLNIFTPSIDS-------------------------------GLAQPYA--------------VMFYIHG------GEFSHGASNL---FPAHILAAFYNV-VVVSINYRLGVLGFLSTG---------DE---NSPGNYGILDQAMALRWVYDNIKAFNG-----NPDAITLFGPGAGAVSAGLL-MVAP-K---TRE--MVSKVIAQSGSALADWGVIIDKYRAQNTSRV-YAEMLGCSI----------ESSWKLVQCLKNGRSFFELGNSELKPHI-------------GMFPWAPV---------------LDTNFTIP---DNWYEDWRASDWRFF-ME----TP---------EES------------------IKNH-------------------RF------------------------------------------RNNLA-YMAGVTTQ**E**AAYLI-YNNVTLA---RNQYIIDIELFEQKIWEF----------------VLQYNYTLN--------------SQGVY---EAIKYMYTYWPD--PK------------NIT-------HIRDQY-------------------------INLLSDFHYVAPFDKMAKL-------L-IEK------------------H--VPTYLYVLNTTVEA----LRL---------------P------------QWRS---VS--**H**DTELLWLT--------GAPFM-DVEFFPQKW--------NLKRD-MWTDNDRNMSHFFMQTYSNFAKYG

>CCE40

EVFLGVPYASPPTGS--MRFM---------------PPGTPQ-H----W--------KGIRMADRLAPVCPQKPPDV-----QDETAALK------RMSQRRVEHLKHLTPFLTGNSEQQSEDCLYLNLYTPTIGIFAS-------------------RN------IATKNRLP--------------VMVFIHG------ESFEWNSGNA---YDGSVLASYGEV-IVVTLNYRLGILGFLPPME--------SG---GRGANNGLLDIVAALHWVQENVIEFGG-----DPGNVTVFGHGRGAALANLI-MLTP-M---ARG--LIQRAILMSGSALSPWAMARDS---VKYTKL-IATELNCPL----------EDNRALIECLKSRS-AEDIVAVGLSAGE-------------YLTTFGPV---------------VD---------G-----------IAI-PK----EP---------SLL------------------MEEHG---------------PNALF-------------------------------------------KTYE-LLVGMADN**E**GGHYF-SHLEEVY-----------GIDGDQRKKH----FRNLV-------RNLYSYHRQ----------------EIF---VTVLNEYTDWTV--STA----------PKPS-------RIARET-------------------------AEALGDALVVAPLVKTARL-------H-AKL-----------------GG--KSTFMYVSEHQQEY----NDH---------------S------------NA-----VL--**H**ENDLVYIF--------GAPLV-ESQ-------------LGPFVG-NYTLADQTLAQTFIEYWTQFVKSG

>CCE41

EVFLGVPYASPPTGN--MRFM---------------PPGTPT-Q----W--------KGIRMADRFAPVCPQRPPDI-----HNETEALR------RMPRGRLEYLQRLLPFL----HKQSEDCLYLNIYSPAIV-------------------------------GRSPMHLP--------------VMVFIHG------ESFEWNSGNS---FDGSILASHGNV-VVITLNYRLGIFGFLPPM---------EN---GRGGNNGLLDLVAALHWIQGNVAEFGG-----DSRNVTIFGHGHGAALVNLL-MLTP-M---ARG--LFQRAVLMSGSALSPWAISRDA---QKFTKR-IAQALDCPT----------DDSRLLVECLKTKA-ASEIVAIEIRAPE-------------YHSAFGPC---------------VD---------G-----------IVV-AR----EP---------SLL------------------MEEHA----------------SAQF-------------------------------------------KSYD-VMFGITSV**E**EYFFI-AASEEKY-----------GIEIDRRDRI----LRTLV-------RNLFSYHQQ----------------EIL---LTIVNEYTDWTR--PF-----------QHPL-------TTLDGT-------------------------AEILGDAMIVAPLMRAANT-------H-AKM------------------S--KNSFVYLFGYMTEH----GDY---------------P------------NRVG---AV--**H**GEDLAYLF--------GAPLM-PL--------------TGHFKS-NFSKNEQALSEAFITYWSNFARAG

>CCE42

EVFLGVPYASPPLGN--MRFM---------------PPGTPT-Q----W--------KGIRMADRFAPVCPQKPPNI-----QNETEALK------VMPRGRYEYLRRLLPFL----QKQSEDCLYLNIYSPARVGAM----------------------------GRSPLHLP--------------VMVFIQG------ESYEWNSGNS---LDGTLLASLGNV-VVVTLNYRLGIFGFLPPIS--------EN---GRGGNNGLLDLVAALHWIQGNIAEFGG-----DTRNVTVIGHGQGGALANLL-MLTP-M---AKG--LFQRAVLMSGSALSPWAIAREA---PKFTKR-IGQALDCPI----------EDNKALVECLKTKP-AAEIIAVEVEAPE-------------YHSAFGPC---------------VD---------G-----------IVV-AR----EP---------LVL------------------MEEHS----------------VAQL-------------------------------------------KTYD-AIFGVTSY**E**AYDWM-SAEEEKY-----------GIEAERRDRI----LRTLV-------RNLFTYHQQ----------------EIL---LTVLNEYTDWTR--PFP----------PHPL-------SILDST-------------------------AEVLGDALVVAPLFRAASL-------H-ARV------------------A--KNSFVYVFGYMTEH----GDY---------------P------------NRVG---GA--**H**GEDLPYLF--------GAPLT-PL--------------HSHFKDGNYSKSEQSLAEAYVSYWSNFARVG

>CCE43

EVFLGVPYASSPTGA--MRFM---------------PPGTPT-H----W--------KGIRMADRYAPVCPQSLPDI-----RNESVALK------KMPAGRLEYLRRLLPYL----QKQSEDCLYLNIYTPAIV-------------------------------GREPIRLP--------------VMVFIHG------ESYEWNSGNP---YDGSILASHGNV-VVITLNYRLGIFGFLPPM---------EN---GRGGNNGLLDVVAALHWVQGNVAEFGG-----DARNVTVFGHGHGAALVNLL-MLTP-M---ARG--LFQRGMMMSGSALSPWAIARDS---VKYTRR-VAKELDCPV----------EDNRALIECLKNRA-VTDILRIDLSPPD-------------HLSAFGPV---------------VD---------G-----------IVI-PK----EP---------SLL------------------MEDHG----------------ASFY-------------------------------------------KNYE-MLAGVARV**E**AYFFL-AAAEEKY-----------GIEIDRRDRV----LRTLI-------RNLYTFHQQ----------------EIL---LTVINEYTDWTR--AY-----------QHPL-------SVLDGT-------------------------AEAIGDVLVVAPLVKAANL-------H-AKL------------------S--KNTYFYVFGYQTEY----GDY---------------P------------NRVG---CI--**H**GEELAYVF--------GAPLV-SH--------------LGHFAR-NFSKSEQAFAEAIMSYWTNFARFG

>Am_GB18290

EVFRGVPYASPPIGS--LRFM---------------PPVSSA-L----W--------HGVKVADKFGPVCPQRLPEL-----SD------------KMPKGRVEYLRRLLPYL----RNQSEDCLYLNVYAPVQAGAR----------------------------DGGGRRYP--------------VIVFVHG------ESYEWSSGNP---YDGSVLASYGGV-VVVTINYRLGILGFLNANT--------DSH-LRSPANYGLMDQIAALHWVQENIGNFGG-----DPRNVTLIGHGTGAACVNFL-MTSH-A---VPDGLLFHRSVLMSGSALSPWALVRGA---ANYALQ-VAKHLNC------------SDSQALLRCLREVP-LNALVSVPVKGLE-------------FAPAFGPS---------------VD---------G-----------VVIDPG----DP---------EDQ--DFTLQVDTINTLNNILLRKD----------------VVAKL-------------------------------------------SRYD-LMIGVVRS**E**AYFAL-TADDAQY-----------GIEADRRTKI----LREFV-------RNTYTYHQA----------------EIL---ATIINEYTDWER--PV-----------QHPV-------NIKDET-------------------------LEALGDANTVAPATRTADL-------H-SQS------------------R--RNSYLYVFDYQSKF----GDY---------------P------------QKPG---CI--**H**GEDLPYFF--------GAPLV-GG--------------LSHWPK-NYTRAEMALSESVILYLTNFARTG

>Tu_04g08480

EAFLGVPYAAPPVNS--LRFM---------------PPVTPA-H----W--------RGTRLVNHYQPVCPQKLPKA-----FDAQGNIKDSPSNENLMRGNYGYFSRIIPHL----SNQSEDCLYLNVYLPLPK-------------------------------GDSDKKLP--------------VMVFIHG------ESYNWGSGNT---YEGTILAAYGNI-VVVTLNYRLGLFGFLPTII--------DG---NTRGNHGLMDIIAALHWIHDNIKEIGG-----DEKNVTLV**G**H**S**R**G**AAFVNLL-MISP-M---SQG--LFDKVILMSGSALSSWAISHRI---EDYAKY-LSKAVNCPN----------YDNILMVDCLRTKS-MVELLKVDLEEDD-----------DFYLSGFGPI---------------ID---------G-----------LVV-PS----DP---------RLV------------------MESVNSSSSINSILANIKKASSSSFSHYSHNSVNTNIHSNGAGNNNNNGNNNDNNVNSNMKQNAFGGNKPYQ-LMFGVTRV**E**APILF-NKDEEKQ-----------GIDLDKRDSI----LRTLV-------RNMVNYYQEVITINIYIELITNSENVIS---LTLINEYTDWSV--PS-----------EHPI-------NILDSL-------------------------IDILGDALVVAPITRAASL-------H-YKKLQSQLQRIKGNDKTIKIEA--PSIYSYVFVYQSES----YGY---------------S------------ARLG---CV--**H**GDELPYLF--------GAPLA-QHYLSKS---------LGHFRL-NYSKPELILSEMTINQWVNFVKFG

>Am_GB18836

MA--------------------------------------------------------------------------------------------------------------------------------------------------------------------DNNSRKHP--------------VLLYIHG------ESYDWGSGNP---YDGSVLASYTDQ-VIVTMNYRLGVLGFLNANMAPQ-----TK---ARVANYGLMDQIAALQWVNEHIALFGG-----DPNNVTLMGQGTGAACVHFL-AISPTV---VRG--LFKRAILLSGSALSSWAVVEDP---VSYALK-LAKAVNCTI---PNDL--FKNNELIVDCLRDRS-LEELMKVNIQPPT-------------FLSAFGPS---------------VD---------G-----------VVIKPD----FQ---------KDLLSYMGPEFQGFGPLPK--KAEHG--------------APITSN-------------------------------------------NKYD-LLFGVTTS**E**ALWKF-AERDVQQ-----------GFEGERRDRI----IRTYV-------RNAYVYHLT----------------EIF---YTVVNEYTDWER--TV-----------QHPV-------NTKDAC-------------------------VQALSDAQFVAPLVQTGDLF---TLRH-TKKPNN--PHIAPIPESEK--EPLPKTYFYVFDYQMKD----GDY---------------P------------QKMG---SV--**H**GEELPFVF--------GAPLV-DG--------------FGHFPR-NYTRSEVALSESIVQFFANFVRTG

>Dm_CG13772

EAFLGIPYASPPVGS--LRFM---------------PPITPS-T----W--------KTVRSADRFSPVCPQNIPIP-----PNGPEALL------EVPRARLAQLRRLLPLL----KNQSEDCLYLNIYVPYETRRQR-------------------RNTDD-TTGEPKTKLS--------------TVVFIHG------ESYDWNSGNP---YDGSELAAHGNV-IVVTINFRLGIFGFLKTGG--------KE---SAQGNFGLMDLVAGLHWLKENLPAFGG-----DPQSITLLGYGTGAVLANIL-VVSP-V---ASD--LIQRTVLVSGSALSPWAIQKNP---LFVKRR-VAEQTGCHG---------DMLYDDLAPCLRTKS-VAELLAVKVDHPR-------------FLVGFAPF---------------VD---------G-----------TVI-SPGA--NPLGSTTLPLGSAI------------------VSTSG--------------IEYANF-------------------------------------------PKRD-LIFCLTSV**E**SYLDL-SAQDLEF-----------GFNETRRDRI----LRTFV-------RNNFHYHLN----------------EIF---AVLKNEYTDWEK--AI-----------RNPL-------SSRDAT-------------------------LQFLSDGHTASPLIKLGYM-------H-SLR------------------G--GRAYFLHFKHKTIE----EEY---------------P------------QRSG---SV--RGEDVPFWL--------GLPM------------------SPLFPH-NYTTQERQIGRLMLRYLSNFAKTG

>Am_GB10066

ETYYGVPYATPPIGA--LRYM---------------PPVTPT-P----W--------RGTKLADTVPPACPQRPPEP-----DS------------SLPRSKRAYLERLAPML----ANQSEDCLYLNLYVPKTPH------------------------------GSTPDLLP--------------TLLLIHG------DSYSWGAGNS---FDGTALAAYGRL-IVVSINFRLGVLGFLKTGP--------KG---SAQGNYGLMDLVAGLHWLHENLGAFGG-----DPDRLTLFGYGTGAALANFL-AVSP-M---VKE--LVERVVLLGGSALSPWAIQRDP---LTVKHH-VAQQTGCPG---------NVEADDIAPCLRLRS-LEDLLSVHLDPPR-------------FTSGFAPF---------------VD---------G-----------AVM-PPPIN-QP-TASSSGL-MPL------------------VPGPG--------------TEFANF-------------------------------------------GDRD-LMLGLTSE**E**AWVNL-TDEDLQN-----------GLNETRRDRI----LRTYV-------RNTYRYHLH----------------EIY---STLRNEYTDWER--GE-----------QSPL-------AICDGL-------------------------LSLLGDGQVAAPLLRLALL-------H-SAS------------------G--GRGYFLHFQL--------GER---------------P-------------------SQ--RGEEVPYLL--------GIPLL-RGEIAS----------ILIGQA-NYTSADENLSKLLVHYLANFVRRG

>Am_GB13939

EVFRGIPYAAPPVGD--LRFR---------------PPISPI-P----W--------DGIKLADSFGAVCPQHFPDI-----SNDTAALL------QMPLGRYQQLKRLYMFL----TNQSEDCLFLNLYIPGSGS------------------------------RGLEAPYA--------------VMVYVHG------ESFEWGTGNI---YDGSVLASAGHV-IVITLNYRLGILGFLRTRPYPDR----TP---GSGGNLALKDIAMGLRWVRENIAAFGG-----DPTKITLIGHDTGAALVNLL-LLAP-Y---GKG--LFHRVVLSSGSALSPWASVHDP---NDLRLK-VGEQIGCST----------ENDEDIADCLRGVP-LRELMAVELPEIR-------------FVPRIGPG-------------LPVDQ--------NN--------------P-----DP---------GLD------------------MERA-----------------SDTF-------------------------------------------IKVP-LILGVSTT**E**SNLDF-NENDIQY-----------GFEEDHRNRI----LRTFI-------RNAYVYHLN----------------EIF---SAVRNEYTDWDK--PI-----------LHPI-------IIRDST-------------------------MEALSDGHTVAPLMRIAFY-------H-ARR------------------G--AKTYFYHFSHQTKE----SGM---------------L------------QRLG---SI--RGEDISYIF--------GLPLV-GG--------------GAFFSR-NYSRQDQTVAEAVLTFFTNFAKTG

>Dm_CG31146

DQFLGLPYAEAPTGN--RRFM---------------PPGAPL-P----W--------QGLKIARHLPPVCPQKLPDL-----SPHGS--------ENMSRARHKHLSRLLPYL----RTESEDCLYLNLYVPHEEP------------------------------QSTPKQYA--------------VLVYLHG------ESFEWNSGNP---YDGSVLSSYGEV-IVVTVNYRLGVLGFLRPSI--------DA---HNIANYALLDQIAALHWIKENIEAFGG-----DNSRVTLM**G**H**S**T**G**AACVNYL-MVSP-V---ASG--LFHRAILMSGSAMSDWAASNQS---LQLTMQ-IAHALECPL---NEHVE-AEDDDVLLDCLRHRR-YQDILHIPTALTQ-------------FSTSLGPI---------------VD---------G-----------HVI-PN----QP---------YKV------------------MGHY-----------------TEHF-------------------------------------------SRYD-LLFGITES**E**SYHTL-AALALEE-----------GLRENERDNL----LRFYM-------QSRFDIRPD----------------LAL---AATLKKYQDMYNN-PIK----------ATNL-------EHRDVV-------------------------LDILSDARVVGPLLQTGMF-------H-ADV------------------N--RRNYMYVFGHNSAT----GPF---------------A------------HLPH---SI--MGEELAFIF--------GAPLA-AA--------------GPFPSG-NYTVQEKLLSEAVMAYWTNFVKTG

>Am_GB18720

DVFLGVPYAEPPVNF--LRFS---------------PPRSPE-P----W--------RGTRESQEFAPVCPQVVPKL-----QDE------------MKPVRYEYLERLLPYL----KNQSEDCLYLNIYTPHQPEG-----------------------------QKTLRKYP--------------VMVFIHG------ESFEWNSGNP---YDGTILAAYGNV-VFVTINFRLGILGFLRPGI--------RD---DTASNFGLLDQIAALLWLRENIAEFGG-----DPNSITLVGHGTGAIFANLL-LISP-VA-NKKG--LFRRAILMSGSALSADAIGKAP---LQITKQ-VAHALHCPT----------TTDSDLAICLRGQD-VDTLLNVKIHKPS-------------YVPAFAPL---------------ID---------N-----------AVI-PD----KP---------YNL------------------MKNP------------------QMF-------------------------------------------DRFD-LMYGVTES**E**KYHLL-SPVDLMH-----------GMSEGQRDAV----LKEHA-------KATHELEAE----------------LIL---SKILEQYGDFSP--GFQG---------EYML-------KNRDLV-------------------------LEALSDSGTVAPLIMAANL-------H-SRA------------------N--PNSYMYVFAHPKAT----QEY---------------S----------GQQRKY---TV--**H**SEELPYLL--------GAPLD-------------------GLRG-RYDIGETLFSEAIMNWWCSFAYIG

>CCE44

EVFLGIPYAAPPVGK--LRFQ---------------PPQPVA-K----W--------DGIRDLESMPPVCVQAFPEI-----PTTPTGSWEEAFQLKISTSRLKLLQRIKPFIE---GNQSEDCLYLNIYAPSSHR-------------------------------SAESRIP--------------VLVIVSTG-----DSYSWGAGNY---VDGSVMAAYTNS-IVVTLNYRVGVLGFLPNSL--------SG---TVSANVGLRDQIEALRWLRLNAASFGG-----DRDRVTLLGAGKAAVLVHLH-MLNP-V---ATG--LFRRAALIGGSALSSWALCHDA---DEQALL-LAKSLRCEA------------GEDPVECFRERS-ADELVQASSKLLV----------PEHLCGPFGPT---------------PD---------G-----------DLV-PH----DI---------YAA------------------TSAYT--------------SRSSSF-------------------------------------------GQHD-LLTGVTKW**E**SYQLF-NDYQRIH-----------GIDAEYKERA----LRTLV-------RNSYFFHQN----------------EIL---FSISNEYTDWTK--SE-----------PSTT-------DILRET-------------------------AEALSDATVVAPLMEVTTL-------H-SHVI------------TLSKSQ--RSTFFYVFAYQSTS----CDY---------------S------------HLYS---CTPDSEEALTFLL--------GMPLL-DH--------------QQSRRL-NYSRQDAQISEFMLIYWNNFLRTG

>CCE1

ESFLGIPYAKPPTGK--LRFA---------------RPQAFG-P-------------VGSFDATGYGSSCPQPMSSP------------------------------LQNFSE----RTDSENCLFLNVFRRTGT--------------------------------NISDKKA--------------VVVLIHG------GGFVWGSSGNV-EYNMAPLAGIGDV-ITVSFNYRLGFLGFGYLE----------D---TDFKNLGLLDQRLALEWVRENIGMFGG-----DPDRVTLI**G**V**S**A**G**SMSVNAH-IVTP-LNTHKKK--LFRAAFMDAGVISGVLINDPGI--AENGTKC-VARTAGCAD------------DENLIDCLREVE-LADLLQRVATC-------------GTKHVRFGPV---------------AD---------G-----------VFL-PK----NA---------EEY------------------LKTK-----------------AGDF-------------------------------------------LKVK-TMIGYARN**E**GSMFA-AMRREKY------------PETMTDEEFIDV-IEEM--------IRAFSFRVE-----------VKGDERMK---KDLLGQYLKKFV--------------------------DPFTAV-------------------------EKFIGDGRFVCPSNAFIEN-------Y-ARV------------------N--DQVYVYRFERLLEG----NYFSF----D--------P------------EVFG---VY--**H**ISPFVHFT--------GTYLG-SSDGNH-----------------EVLDADKEFMLEAMRVLSTFAKED

>CCE2

ESFLGIPYAEAPIGE--LRFA---------------LPRAFG-S-------------VGDFEATKYGFSCPQPNVFP------------------------------LQQFSN----TTDSEDCLFLNVFRKAGT--------------------------------TSSDMKALWNGKKRSSFFPIQVVVLIHG------GGFLWGSGGHV-EFNMAPLAGIGDV-VTVSFNYRLGFLGFGYLE----------N---SEFKNLGLFDQRLALEWVQENIAIFGG-----DPERVTLI**G**V**S**A**G**SMSVSTQ-IVTP-LNSQKGN--LFRAAFMDAGVISSTLINEPRV--AENRTRC-VARAAGCTG------------DENLIACLRGLE-LADIQRSGETADC-----------GPIILRFTPT---------------SD---------G-----------IFL-PE----NT---------QEY------------------LKSN-----------------AGNF-------------------------------------------LKVK-TLIGYAQN**E**GSMFA-AMSGAGH------------PTTLTDEDFLDV-IQEI--------IRAYRLRVE-----------IDGDERLK---DAILENYLREFV--------------------------DPFTAF-------------------------DQFIGDAFFYCSSNSFIEN-------Y-ARV------------------N--GHVYVYRFDKVMER----KYFSF----D--------P------------KIFG---AY--**H**MSPFIHFT--------GAYLE-DSEGER-----------------RVADADQEYMLEAMKVLSDFAKSD

>CCE3

ESFLGIPYAKPPLGD--LRFA---------------YPQVFG-P-------------VGELNASQFSSKCHQRESKS------------------------------TSRPAS----AVESEDCLYLNVFRKAGT--------------------------------QADAKKA--------------VLFVIHG------GAFTEGSSSDE-SYNAKPMVALGDV-IVVSINYRLGIFGFADMK----------D---LAPGNLGFFDQLLALEWVHENIAAFGG-----CPHRVTLV**G**V**S**A**G**SISVAAL-LASP-LV-RGKN--LFRGVFMDAGVMSRTSIMTQDY--TLNRMKE-IAGKVGCG-----------TEGGKMLSCLRNVN-ATRLIDLSSDLE------------TSSIFTFIPT---------------VD---------G-----------KFV-PV----DP---------TKY------------------VQEN-----------------PDKF-------------------------------------------PDVR-MIIGVTKD**E**GTMFA-AMVPEAQ-------------KVETETEFVAL-SEKM--------SKNLVYPLD-----------FD-KKEVK---AAVTQTYFGKSV--------------------------NHFHDI-------------------------SDFITDGTFACPTDHFVKE-------Y-SSV------------------H--KNVFVYRFERTMKT----KGILPGMN-D--------P------------ELLG---AF--**H**GSPFIHMF--------GSCLA-FSGE-----------------T-PLDADDKQFSMDSVKMLADFAKSD

>CCE4

ESFLGIPYAKPPVDE--LRFA---------------HPQAAV-P-------------FGELNATEYSPTCIQTKIFP---------------------------------IRL----QPSSEDCLYLNVFRKAGT--------------------------------RPQSKRP--------------VIFVIHG------GAFCVGSASQA-FYSGLPVTATGDV-IVVTINYRLGILGFADMK----------D---LAPGNLGLFDQLLALEWVHDNIASFGG-----DPERVTLL**G**V**S**A**G**AMSVSAL-VNTP-LI-RGKN--LFKQAIMDAGVMSRTTVMTQDI--SFRKVKD-IAAKIGCGT----------AEGERMLSCLREMN-ATLLTDASSDPE------------SSPILTFTPT---------------VD---------G-----------KFI-AL----EP---------SKD------------------TQEK-----------------SDKF-------------------------------------------ADVR-MIVGVAKN**E**GSLFT-GFYPAVK-------------TLKNETDFIEL-AKEI--------SRGFLYPLT-----------LE-TEDAK---DSLIRTYFGKSA--------------------------DHYEDV-------------------------AELIADGTFICPSNAFVKN-------Y-AKT------------------H--KNVFVYNFEKVMKR----KYLKFG------------P------------DNVG---AY--**H**GSPFANFF--------GSYLV-TPKED--------------IDG-PLEPEDVQFAKDSVELLVNFLNFE

>CCE5

ESFLSIPYVEPPVGL--LRYE---------------KPQEYG-L----F-------PELKLNGTAYPPACPQIVGSL-------------------------------AQASN----ISQSEDCLYLNIFRRRGT--------------------------------NLNDQKA--------------VLVIFHGGLFLQPGGFSHGTSADP-HQNPEALVAAGDV-ILVTFNYRLGVLGFADLK----------E---FAPANLGLHDQRAALAWVKENIGEFGG-----NANDVTVMGTGAGSMSIVAH-IMSF-V--DNRD--FFHTAILDGGVLSSNALLEDTT-SSFRRISK-IAYDLGCP-----------LNSRDMIECLQDSD-LEDLISLSEEYMP-----------ANGLAAFVPT---------------AD---------G-----------FII-PT----RP---------DAF------------------IDDN-----------------NVLL-------------------------------------------KKVR-LMIGFAED**E**GTLFT-NYTKSTF----------NFTEDKTEDEITNY-CLKL--------SDAYNYPLD-----------VR-DRLTK---DTVKEIYAARHEK-------------------------WPRRAV-------------------------VTFQGDGVVKCPTNNFIKQ-------L-CRR------------------T--SEVFVYHFERKFSK----NYL---------------P----------EMEDLG---VF--**H**TSPFQHFV--------GSLFL-YVKP-----------------G-DMAPEDRRFMLDTMKMITDFVADQ

>CCE6

EFFLGIPYAEPPVGE--HRFA---------------LPRAFG-A-------------VGEVEATEYGPSCLQPSSGP-------------------------------LAPEN----RKLSEDCLRLNVFRRQGT--------------------------------NVNDKKA--------------VIVTLHG------GGFVAGSSSDP-LQNTAALAGLGDV-VVVSLNYRLGILGFADMK----------E---FAPGNLGLRDQRLALEWVQDYIADFGG-----DPGRVTVM**G**V**S**A**G**SMSIAAQ-IITP-I--DERN--LFSSVVMDAGVVASNGFHEDSD-SSYSRLTK-IAEQVGCP-----------LHSLEMVDCLRKIE-AERLIAFSLNTTG-----------DNGISYFVAT---------------LD---------G-----------DFI-PR----EV---------EDY------------------VREN-----------------SSNL-------------------------------------------RKVR-TIIGYARD**E**GSFFV-SSKFMAK----------DHPNPESTDEVMAY-MELL--------SQNYDYPLN-----------FG-EEKTR---ELLSRLYIERHPN-------------------------DSLAAI-------------------------ASLQSDGIFKCSINNFIRS-------Y-TQY------------------N--EEVYVYQFERELKS----VYAKIL---D--------P------------NILG---AF--**H**LSPFLHFC--------GSLFL-GPE--------------------PLHPDDRTFILESIDLISRFAKAV

>CCE7

EAFLGIPYAKPPLGE--LRFA---------------KPQPFG-S-------------VGNFQATELPPLCPQLKMCP---------------------------------LGR----YSVEEDCLYLNVYRKSGT--------------------------------KKDDKKA--------------VLAIIHG------GGYIVGSSADV-FQSAEPQAGLGDL-IVVSLNYRLGIFGFADMQ----------E---IAPGNLGLQDQRLALQWIQRNIAAFGG-----DPKKVTVM**G**G**S**A**G**SMSIAAQ-IITI-V--DREH--LFRAAVLDAGVVNSNGFFESSE-SSFARVRK-IAEIVGCP-----------DAPDEILACLRNVP-AHYLLANSTETTG-----------LGGITSFVPT---------------TD---------G-----------VFL-PK----DI---------GEF------------------LAQE-----------------SPSL-------------------------------------------RKIP-MIIGHARD**E**GTMFV-SLADRNF----------NFTIDHTKDEIVDY-CAAI--------GQKFDFPLN-----------AT-QSEIR---EKIGRIYVDENSG-------------------------NSWKAA-------------------------AAFAGDGIFACPINSFIKN-------Y-SRH------------------S--DKVFAYRFDRRLKD----THFKIF---D--------P------------SLLG---AH--**H**FSPYLHFS--------GALFL-DDAD-----------------G-PIDGGDQRFSLDAMNMIASFAKHD

>CCE8

EAFLGIPYAKPPVEE--LRFA---------------RPEPFG-P-------------VGNLEATELPQQCPQFPMHG---------------------------------SGR----YSDIEDCLYLNVYRKRGT--------------------------------TPEDKKP--------------VLATIHG------GSYSAGSSADV-NQSAKSLVGFGDV-ILVSFNYRLGILGFADMK----------G---NAPGNLGLHDQRLALKWIKENIADFGG-----DPEKVTLM**G**D**S**A**G**SMSVAAQ-IVSI-A--DSDQ--LFHAAILDGGVVASNGYLEDSE-SSFTRIKR-IAEIVECPT----------ECSVGMVTCLRSLP-VAALLANSTTTYG-----------STRISMFMPT---------------TD---------G-----------VFL-PE----DV---------QAY------------------VANA-----------------SSRL-------------------------------------------PKIP-IIIGYSLD**E**GSFFL-GSVGDRE---IEGSN-FDYTREYSRSEILDF-CSNI--------GATFNFPFN-----------AS-QGDIR---EKIGKVYVDENSG-------------------------VAKEAL-------------------------SAFLSDGLFKCPINAFIRD-------Y-SRH------------------S--DKVFAYQFDRKLKK----TYFKLL---D--------P------------SVLG---VF--**H**YSPYLHFA--------GVIFL-DEG--------------------PVDEEDRKFALDAMDTIANFIKSA

>CCE9

ETFLGIPFAKPPIGE--RRFA---------------RPHPFG-A-------------FGELDATKHSPVCVQSIHAL---------------------------------------GANSSEDCLYLNVFRKEGPL-------------------------------YQTAELP--------------VIIVIHG------GAFASGGASTF-SFSGVPLAAHTGA-VVVTIQYRLGIIGFAQSD--------------TIPANLGLQDQRLAIQWVHDNIASYGG-----SPERVTLM**G**P**S**A**G**SMSISSH-IMSP-EL-RDRN--LFQSVIMDGGVLGGIVVPKSET---VRRLVR-INAKLGCP-----------LSGQPALRCIRDVK-VQDLIEYSIESPK-------------AATTFIPT---------------DD--------ES-----------DYR-PK--NKGP----------------------------------------------------GQF-------------------------------------------APVR-MLIGTAEN**E**GEIFV-KTRIDEK------------AEMRNFEDFLVL-CREL--------RDLFDVKVD-----------ID-NPDTR---ELLRKSYYEKHE--------------------------NFRIAA-------------------------AEFVGDSVFVCPVNEFVER-------F-SKF------------------N--GEVYVYRFDRQLAQ----TYKKL**H**L--D--------P-------------QGG---AK--HWHPYVHFS--------GSLLT-LGP--------------------FAHSADVKFSLDSLKMISDFVA-G

>CCE11

EEFLGIPYAEPPIGE--LRFK---------------KPVPLQ------L--------KGTLNASSYAPGCTAEFGMF--------------------------------TTET----FNFSEDCLFLNLWRPKGT--------------------------------TAKDSKA--------------VVVVFHG------GGYVAGDGSEH-DWRGGSLSALGDV-IVINFNYRLGVFGFLDLG---------IE---EAPGHQGHWDQLLVLEWVRDNVRNFGG-----DPERVLLI**G**V**S**A**G**SFSISAH-LMSP-H---SRG--LFHAAVLDAGIVALGDTIED----SAERATK-IANSVSCSN----------STVEEIVECLKNLD-ASLLLELQQEFA------------VSQTYVFRPT---------------QS---------N-----------EFI-GD----GSL--------EEL------------------LATA-----------------AENF-------------------------------------------NHVP-IIIGDSAK**E**GAFLM-TERVNEP-----------LPELATLEQTLGL-MRNICEWHFV-------------------------PEFPV---DSIMSAYELSDD---------------MDTF-------AYRNAT-------------------------ADFIGDSLFVCPVTKFAAV-------Y-SKY---------------------SMVYHYFWERKTVS----STV----GAD--------P------------WAFG---AY--**H**GIPFYHML--------GSFFE-SFD--------------------DLAPEDVSYSMAAIEMLVEFAKTA

>CCE12

EAYLGIPFATKPVGK--NRFREA-------------SLRPVH-T----L--------GAEFDATQWKAGCMQQPGLF--------------------------------TNDS----NLFSEDCLYLNVWKRRGT--------------------------------ENGDLRP--------------VVFIIHG------GGYVAGDGHEF-DWRGPQMAFFEDL-VVVNLNYRLGVFGFLDLG---------LP---HAAGHQGHLDQVLALRWVKQNIRYFGG-----DPDRVTLF**G**V**S**A**G**SFSISWH-LLTG-L---SAG--LFHAAVIDAGVLTLTHAETKRD--HILRARQ-MVQASVCPN--LRNDSS-KDQRREILDCLMGMD-AKRLVELQNEYG------------VSQTYTFRPT---------------FN---------NE----------KYL-PR----SP---------SCM------------------TNEK-------------------AF------------------------------------------AVNVP-IIIGDTTN**E**GLFLL-SKKVHGP-----------LPSFQNFEQVLEW-DINILRGH--HAADAAQYGPEN---------------------ETIRDIYHGRDG--NRS----------SAGE-------TLFNEA-------------------------TQIIGDALFVCPVMNFANR-------Y-STV------------------Q--PNVYFYRWQRLRVD----ETF---------------P----------GEREKG---AY--**H**GLMFYTGAG-------GQYLS--------------------LYG-QMADADKLYIQKTIRMIADFA---

>CCE13

DVFNGVPYAESPTST--GRFR---------------KSSPVK-R----F-----TADGSVYDATHYRPGCIQPQGLF-------------------------------GINES----TVFDENCLFLNIWRRSGT--------------------------------DDSHRKP--------------VVLIIHG------GGYTAGDGHEY-DFRGTQLAAFGDV-VAVNMNYRLGLFGFLDLD---------LP---EAPGNMGHTDQTLAMEWVQKYIALFGG-----DPQRVTLF**G**V**S**A**G**GFSISAH-LISK-Q---SEG--LFHAAIIDGGVITKAKTDSRAE--HLTSVRK-VARGLNCSD----------ETTEDLLKCFTEAD-AQKLLEQQQANSLLK--------------IFTPPT--------------HG---------N-----------GFL-AN----IP---------EKI------------------VNHQ-----------------PGSF-------------------------------------------TRVP-ILVGDCSD**E**GSIVF-YPEIENV-------------NLTSVSETLNY-LQTELGDH--PLSRNFK-----------------------R--EDIVEGYNISEG---------------GTSE-------YYRNVS-------------------------LDFVGDGFFVCPVRNFVHH-------Y-SSV---------------------ASTRFYYWEHLTKQ----KVS-----LD--------P------------LTWG---AF--**H**GTPFFHMI--------GSQFT-YFEND------------------TISSEDADYVKKSIRMVVDFA---

>CCE10

EVFRGIPFAEPPIGM--RRFR---------------ETQLVE-K----F-------PYSPFNASEYPPACIQIYAN-----------------------------------------FTQDEDCLYLNIWRPAVR----------------------------------RTKLP--------------VIVMIYG------GAFIVGHSRSP-NYSGLPLTALHDV-IVVNFNYRLGAFGFSDLSE--------IV---NLPGNQGFLDQRLAIKWVKEFIEDFGG-----DPTRITLM**G**C**S**A**G**AVGVSYH-LKSP-L---SQG--LVRGAVMDAGVLSTKRLETVRS--SLRRMIR-LAVKVGCNS----------TRPHQLLRCLRKVP-AAVINDASMTVM------------DSPLLSFRPT--------------------------K---------------------FP---------EEL------------------LEAV---------------------------------------------------------------SGNVS-VFLGHSGS**E**GLTLV-SNGFGIN---------DPLPEFRSTHEAVEW-CKVLLMSG----LREFEENIE-----------SQ-WSQFK---DFLDRLYFKGYEH-------------------------SLVDRA-------------------------ALFLGDMYFHCSMLSSYKT------------------------------T--PVSTFYVFERAFKK----NVF---WDND--------P------------EIYK---SF--**H**MTAHMHFV--------GSQLL-TLRD-------------------EIHPEDLAFTLKSMEWIADFASHS

>CCE14

EVFAGIPFAKPALKL--NRFR---------------PPEKLK-R----SP---LISRFAPFGVIKYPASCVQ--------------------------------------SPA----TNTSEDCLYLSVWRRQGT--------------------------------IAINRKP--------------VVVIIHD------GGFYRGSGHG--EWHGNALAALGDV-IVVGFNYRLGVLGFLDLG---------VH---GAEGSVGLEDKQLALGWVHDHTSGFGG-----DRHNIILLGTEAGASSAIYH-INQ------TSS--RYQGAIVD---LLWS-PPDDKR-TLFERGLE-VSKNLNCSG----------GEPEAIAFCLRTQS-VETLAGIGQI--------------------FHPN---------------IS-----------------------------------------------------------------------------------IDVRT-------------------------------------------NGLP-VVVGFAVPGSRNVA----------------------LASVCKTLRW-AESA----------------------------------------------------------------------------------------------------------------------------------------AEA------------------G--SSVNLFLWRKNSAA--------------------------------------------------LLEFI--------GSALG-SRSGDSES-------------S-QDHNNPAIPRLNLTETVREITQ--

>CCE15

EVFAGIPFAKPALKL--NRFR---------------PPEKLK-R----XX---XXXXXD-------------------------------------------------FELPA----TNTSEDCLYLSVWRRQGT--------------------------------IAINRKP--------------VVVIIHD------GGFYRGSGHG--EWHGNALAALGDV-IVVGFNYRLGVLGFLDLD---------VH---GAEGSVGLEDQRLALEWVHDHISGFGG-----DRHNIILLGTEAGASSAIYH-INQ------TPS--RYQGAIVD---LLWS-PPDDKR-TLFERGLE-VSKNLNCSG----------EKPEEIAFCLRTQS-VETLAGIGQF--------------------FYPN---------------IS-----------------------------------------------------------------------------------TDVRM-------------------------------------------NGLP-VVVGFAVPGSRNVA----------------------LASVCRTLRW-AESA----------------------------------------------------------------------------------------------------------------------------------------AEA------------------G--SPVNLFLWRNDSPA--------------------------------------------------LLEFI--------GSALG-PSSGDNKS-------------T-QDHSDSAISSLDLIEIVSSFARDH

>Tu_11g05770

YQFLGIPYAKPPVDK--LRFQ---------------LPVEPD-P----W--------TEVEPATAPGPACLQPPVLP----------------------------------NI----KETSEDCLYLNIFVTESTFN-----------------------------NSSNKQRP--------------VLFWIHG------GGFTTGSG----VIDGLPIVSLHDV-VL------------------------------------------------------FGG-----DPNSVTIF**G**A**S**A**G**SVSVSAH-LLSP-L---SKG--LFKRAILESGTIYQNH---NDD-TLVLDSWK-LYNKTSCSK------------SKDVLECMQNLS-ATEILNNISD----------------KVLAYSFK---------------VG---------D-----------DFL-PY----EP---------SKA------------------FAEG-------------------FF------------------------------------------DNSID-ILLGVVKN**D**GSVFM-KAIDPIV---FDYS---KVPQPISYQQATTY-LKRIF------------------------------NSESV---DYFRELYFGPET----------------NDS-------DFRSQL-------------------------EIAYSDATFICPTYVFGLQ-------Y-ASSLGSQ--------------G--GKVYAYYHTQKPKT----DHM---------------P------------EWIG---TY--**H**GADVSYVF--------GTNLQ-NP---------------------GNSHKDASLSREMMKIWTYFAENG

>Tu_29g00970

YQFLGIPFAEPPLNE--LRFQ---------------KPVPKK-P----W--------NGVLSMNKWGSACMQPIFPGF----NTE--------------------------------LPISEDCLILNVFTTEAAF-----------------------------------------------------------------QDRKNGKKNKL------------HV-IIVSLNYRLGSLRFLQLP---------EA---GVPGNMDLWDQQLTLQLGKYHIIHFGG-----DPDELTIFGEPAGSMSVSAN-LVSS-K---SKG--LFKNIFHYPKS---------------------FSSKIGCTS-------------NDYKSCLSNCQ-FNQFPAADE-------------------LKFWPT---------------VD---------S-----------EFF-PN----HP---------EEL------------------VSNH-------------------GL------------------------------------------DQRIN-VLLGIVGN**E**GTMLC---------------------------------------------------------V-----------------------------------------------------------------------------------------------------------------V-------------------------SNVYGHVQTQRSSH----AVL---------------PV-------SNQAKWMSNV-AS--**H**ADTFRMVF--------GHPFT-KHD--------------------KLKNED---------VVCHF---S

>Tu_01g10830

EAFLGIPFAEPPLGH--LRFQ---------------LPKPLN-P---TW--------STPYEAVNFKDACIQRRESYFN--------------------------------------FNISEDCLYLNLWRPESAG------------------------------KHDGELLP--------------VFYLIHG------SGYIKKSGALP-QNQGDVLAATQNV-IAVNSNYRLGALGFAHGS---------RT---SMPGNIGIYDTLIVLRWIRDNIRSFGG-----DPNRVTLA**G**Q**S**A**G**SNMVTII-ATLPNKF-ETQH--LFSRLIMMSGVTINHMQADHVS-TALSKTRL-LATKVNCLSRFDQFESS-GELSDETVDCLKKAD-AYDLLEAQFSEDVIA-------LGNVEVAAFLPV---------------YG---------T-----------ELI-TD----HPM--------INH------------------IKRI------------------ESI-------------------------------------------KDKP-MLFGVEQD**E**TTRYV------------------SLSNISTMNDA-----YEFIK------KRITKAIKGI-------------KTDKI---DYFYKFYFDDVD--PT------------NSD-------EIKAKC-------------------------VNFFNDMCFHCPSLILAEF-------F-SQK------------------S--SNIHFYLNGYVGEV----------LAQD--------PG-----------RPYG---TL--**H**ADDIMFAL--------GEPIR-TYK--------------------NYTENDRKFGQLFSNLFGDFIK-G

>Tu_01g10760

ERFLGIRYGLIP-----RRFV---------------HSQVNNHS----W--------TGILNATKYGSICPQDPSD-----------------------------------------LPMDEDCLFINIWRPAMSG-------------------------------TNKRLLP--------------VLFWAHG------SGYQHGSGAQN---SGDLLAPYGNM-VVVTFNYRLRSFGYAFGD---------EN---EIQGNQAVSDIISALKWVHQNIAFFGG-----DPSRVTYA**G**H**S**A**G**SMMGSIIPVLTA-L---DDS--LYSQLWLTSGVCVTPMYIEDTS-VGLAKTKL-LASKVGCGS-----DAP-GPLTSQTISCLQTVD-MSTILEHDSGSDIKK-------IGGADDPPFLPV---------------YG---------T-----------PLI-PK-----PL--------VEL------------------FKSH------------------PRI-------------------------------------------RKTT-ILNHIHQD**E**EGLLY------------------SDLKPTNISEAY---------------KMAFDHLAFVK---------KDLTKAQM---QPFFEYYFNETN--DS------------DPK-------ALKTSL-------------------------TNFLTDYTWGCTSLMLAEL-------Y-STY-----------------HSVRFGVFTYNLDKYGKE----------------------A------------RPNG---PY--**H**GDEIELFF--------GEPFY-NNPYSSKVGKGDF----------DYSMKDKIESLRLMKSLVDYVH-G

>Dm_CG5397

YSFLGMHYAEPPVGP--LRYS---------------RPVYKR------L--------AGDFNATKHGPPCIQPHPQF-----PQR--------------------------------IIGDEDCLLLNVYTPQMP--------------------------------DETTGLP--------------VFVWIHP------GGYRYGSAAQ---YDATPMAQRGA--IVVAPQYRLGSLGIMGDG---------TK---QFDGNLAMFDLAAALRWVTDYISYFGG-----NPKQVQAIGHGSGAASAMYL-SMSP-T---SRSAGDVHGVVAMSGTALSQYAMDKEP---VQSVQE-VAKINGCPT----------GNELEIVNCLRSKS-AEDIIKNDDKVQTERLAGRALVKGLTGNVGFQPH---------------IESEDD-----G-----------RAL-PSLIVGEP---------EQQ------------------LKSS-------------------NF-------------------------------------------SGIP-LLTGVTKH**E**TANSVTVETIEKV---FGSAEQFLGSLSDSLNKL-----TSFLKI-----DKLTGQIAKP--------ELPGLTSVLT---PTLQDVWKVPQA---------------LNVD-------QVLSKV-------------------------VESTTDVLFNLPAVLTTQV-------W-SRL---------------------APAFMYSFEYNGTKSKGINFLKGLPIVSET--AHDKP------------ETVG-------**H**GDEIGYMFDANDIF--GNPME-ETR--------------------LTSAEDLKVRNNLIDLLVKFANKD

>Dm_CG9280

NAFLGIRYGTVGGGL--ARFQ---------------AAQPIG------Y--------QGRVNATVQSPNCAQFPELD----------------------------RLRLSESR----GENVDDCLTLDIYAPEGA-----------------------------------NQLP--------------VLVFVHG------EMLFDGGSEEA----QPDYVLEKDV-LLVSINYRLAPFGFLSAL---------TD---ELPGNVALSDLQLALEWLQRNVVHFGG-----NAGQVTLVGQAGGATLAHAL-SLSG-R---AGN--LFQQLILQSGTALNPYLIDNQP---LDTLST-FARLARCPP---PSINPSAQGLKPLYDCLARLP-TSQLVAAFEQLLLQNEHL-----GLTQLGGFKLV---------------VG---------D---------PLGFL-PS----HP---------ASL------------------ATNS-------------------SL--------------------------------------------ALP-MIIGATKDASAFIV-SRIYDQL----------ARLQSRNVSDYI---------------DVVLRHTAP-----------PSEHRLWK---QWALREIFTPIQ--EQ------------TAS-----LQTVAPGL-------------------------LELSNYILYRAPVINSISQ---------SYR------------------S--VPAYLYTFDYRGEH----HRFGHLSN----------P------------LPFG-VDAS--LSDDSVYLF--------PYPPE-AS---------------------RLNPLDRSLSRALVTMWVNFATTG

>Dm_CG9287

LQFVDVRYAEPPTGL--HRFK---------------APRPIE-P----W--------EDVMDATAEKIGCPSVVSMD-----------------------------SLRRLDD----VLDVEDCLTMTITTPNVT-----------------------------------SRLP--------------VLVYIHG------EYLYEGSNSEA----PPDYLLEKDV-VLVTPQYRLGPFGFLSTK---------TD---EIPGNAGFLDIFLALQFVKHFIKYFGG-----DPSRVTVAGQVGGAAIAHLL-TLSPVV---QRG--LFHQVIYHSGSAIMPIFLEEDP---RKHAQE-IAKKADCKM----------VTVRDLNTCLMELT-ALELLTAFMEHALEKSDL-----GIGHTGGIQFT---------------IG---------G---------PSGVL-PK----HP---------YDL------------------MLET-------------------NF--------------------------------------------SYP-AMGGCPKNAGSRVL-NEIVDND---FEGK--IPDDEYNTYNYI----------------DHVIRQTVG-----------TDKTMLLT---SFVTHDFFNRNL--ME------------NGT-----FDTLIPRL-------------------------IDVAGTLNHKLPVLLALNM-------N-NKH------------------N-PDNTFLYSFDYAGEF----NRYKEMDEETNL----QSP------------FKAG---VS--LTDEALYLF--------PYPEH-VT---------------------RLSRPDQSMAHRMVELWTNFVISG

>Dm_CG9289

MQFLDIPYGKA------ERFR---------------PAEPAP-S----W--------KGVLPAHRPHAGCPSIQDLIVF----------------------------AKLEED----GFDVEDCLRLSVNTKAME----------------------------------GKSLP--------------VMVYIHG------DFFYDGDSVEA----APGYLLEHDV-VLVSVRYRLGPFGFLSTL---------TD---EMPGNAAVTDIILALKWVQQHIASFGG-----DPQRVTLFGQVGGAALVNVL-TLSPAV---PAG--LFHRVIYQSGTALSPAFITDAP---LGATKA-IGRIAGCKQ---------STKVDQLNKCLNRLN-ATMLLAAFSVHGENQPSL-----SGGAYGGVQLV---------------IG---------G---------PSGIL-PE----HP---------GRL------------------LAAE-------------------KF-------------------------------------------QAYP-TMGGSVKHGGTFML-RDIFADI---FNET--ILDDTMTGRQYI----------------DTIIEQANG-----------ADPSGSWK---EFSDEEIFTQND--VK------------NGT-----FKRLTPGL-------------------------IDLCSTISLKNPVLLVLQA-------N-AKK------------------L-PNSTYLYSFDYEGEQ----NRYATGEDEANF----V-P------------FDMG---VS--LTDDNLYLF--------PWPRF-LA---------------------LNSNRDLKVARRMVALWTSFATTG

>Tu_02g06930

TSFIGIPYASPPVGS--GRFS---------------NPKEPV-K----W--------SGFLDATNRRVMCPQVEQRF-----------------------------RLESDPQ----IGESEDCLYLNVQVLSRG--------------------------------PVTANAP--------------VVVYLHG------GEFKYGGKDFY--YLDVLPQNLNSL-VFVTINYRLGPLGFLSTD---------TN---EVHGNYGLRDIIMALQWVQSEISQFHG-----DTNSVVLMGHDAGAIAANML-MLST-Y---NKV--YFNAVVCSGGTVFTPWAFEHQP---STISRE-LALSLGCGY-------------TQIRRCLQQKS-TSEILAVARTKK----------------LHFRPT---------------MDRNLS-----D-----------PLF-HD----IP---------QIQ------------------IENG-------------------FF-------------------------------------------ARVP-LIVGITEQ**E**GALDY-YRNYRQI-----------QAISKIQDKI-----RYLI-------GPFVKRFAN--------------LDVLA---SLVHYQYFPDEE--ARSGRQITTVGRGYNQG-------QVDERLVQTFYTPYQLKKRCNFTPVTILYQNFTFTKTNSLALPLECFREIWHLMLPLINSEPL-----------------T--WTIYLYCLKQFIQN----NTLRLTRQQILFSLAFYQP-----LK-TRSKLVTG---KI--TKRQLALTF--------ALPVK------------------------------------ALLMVACFVLSV

>CCE35

DAYFGVPYAEKPV-----RFA---------------RAKEIR-A----W--------TGVRTAFVARDPCPRRNPGI------------------------------------------VIESCLYLNVLKPRG-----------------------------------SESLP--------------LLVWFLG------DGMN-GEEDSL-RMNFSALAATENI-VVITVSFRVGVFGFLNAS---------LD---DIQGDIGIHDQRLALEFINENVEAFGG-----RRDKITIGGFGFSGSIAGMH-LLDE-T---ASH--LFQRIVLLSGTPF----MLNRK-TFRNPLHE-FLRVVRCDA----------LDPVETVECIRMKH-TSELHSGL---------------------NFGPN---------------SG---------GG----------TNL-PG------------------------------------YTSG-----------------NAKI-------------------------------------------RGQQ-ILLGSYAYSGWKDD-TDIREFV-----------SSRDPDSDRL-VM-LKEAV-------ELCFDSPMAS------------------VENRNIFRAYAPSAP----------------NNLQGKNFGPEILRLI-------------------------GEMRQDLLHRCASYFWARN---------SGL------------------A--NEIFVFEIEFS-----------------------------------------------------------------------------------------------------------------------

>Dm_CG12869

YAFLGIPYAQAPINE--LRFA---------------PAKPSS-S----F--------NRTLQATTMQPLCPQLANTIYDE--SSD----------------------GSMPRS----VSTDEDCLYLNIWTPESG--------------------------------MRYGKLP--------------IVVIVTG------EEFAYDWPRN--RINGLDLAGEG-I-VVVSVQYRNNIYGWLSLG---------EQH-RNVPGNYGLSDVQMALRWIRRNADAFGG-----NPDHITLLGHGSGGAPLALV-ATLE-D---SSQ--VKQLVLMSPGPIMRALGQNHQK-WIVETGQV-LVQKLGCQFE--------EAQRRQLMGCLRRKS-REDLLRAYESVYNHGNGSSQL---------------------------------------G-----------VIL-PE---GLPL--------EQR------------------LRNK----------------------------------------------------------------TLPP-VLLGITSN**E**GAFLQ-DYWLDVA-----------REGQVALHKYI---NHTLL-------PNVMRALESV----------GEESSTQL---AAIRWRYFNGKG---------------EGVS-------HLLAGM-------------------------QRLLSESLYELPYSRILEL-------L---------------------NG--TTSYAYVFDHSHSM----DMR------------------------GRRNLFGG---AS--**H**SSDLPLLL--------GPSLF-----------------QQIARR-RFSGEEEQLCRKIRGAFANFIKNG

>Dm_CG9704

FAFRGIPYAKPPVDR--LRWK---------------PAELID-DINMCW--------NDTLQTHNSSVVCTQRLGNG---------------------------------------TTVGDEDCLYLDVVTPHVRY---------------------------------NNPLP--------------VVVLIGA------ESLAGPSPGI--LRPSARYSRSHDV-IFVRPNFRLGVFGFLALDALTK-----EAH-PPTSGNYALTDIIAVLNWIKLNIVHFGG-----DPQSVTLLGHRAGATLVTLL-VNSQ-K---VKG--LYTRAWASSGSAILPGKPLSES---GKQNEQ-LMATLECAD----------------IQCLREAS-SERLWAATPDTWLHFPVD-------------LPQPQEANASGSRHEWLVLD---------G-----------DVV-FE----HP---------SDT------------------WKRE-------------------QA-------------------------------------------NDKPVLVMGATAH**E**AHTEK----------------LRELHANWTREEV-----RAYL-------ENSQIGALGL-T-------------------DEVIEKY---------------------NA--------SSYASL-------------------------VSIISDIRSVCPLLTNARQ------------------------------Q--PSVPFYVVTQGEG-----------------------PDQL----------------AT--VDADVQAIL--------GRYE-------------------------PHTVEQRRFVSAMQQLFYYYVSHG

>Am_GB19830

FAFRGIPYAVPPLEN--RRWQ---------------PAESLR-KIEYCW--------KNTYQAHNSSKVCLQRETSG---------------------------------------RIIGTENCLYLDVFTPEVRY---------------------------------DLPLP--------------VVVMIGA------ETLSGGSPGV--MQPSAKLARVRDM-VFVRPNFRLGIFGFLATEPLSR-----TSH-PLTSGNYGLSDIIAALQWVHLNIENFGG-----NKSSVTLWGHRAGATLVTTL-VGIR-R---TRD--LFQRVWISSGSAIFPGRELEFS---ETLNEL-FLNSTRCND----------------AACLRSKS-AEEIMDSVPETWHLGNIG-------------LPEAREATIRDRRHEWLVLD---------------------RAI-LQ----EPV--------GQI------------------WARD-------------------EF--------------------------------------------SVK-IVMGTTAHAGAPLK----------------YLISNITLNSTQV-----EKIV-------KESLLGTTGLA--------------------DEALRRY---------------------NAT---------LKGL-------------------------LSMISDIRVICPLLTVARM------------------------------K--TNIPFYVATQPRGQ-------------------FADP------------------------DSDAAAIL--------GSYA-------------------------ARTPAEKRHVSAMQQLFNHYVWHG

>Tu_01g14090

-IFKGIRYGVAPIGS--LRWS---------------STQPI-------WVDSAYCDAKSQKRATKFGPSCFQINPYT----------------------------------KK----YQGSEDCLFLNVWTPTLND---------------------------------SANLN--------------VMVWIHG------GFLQFGSGHDPGLRPSAHLAVSMNT-VFVSLNYRLYALGFLNLADLPSVRANDLK---SITGNYGLSDQLVALQWIQRNIAKFGG-----SPQKIITFGPDAGAASILAH-LTNP-K---ASQ--YISKAWLIGPTLFLNRTA-----DSLNHIQH-FVNRTGCEN----------------IDCLRNLS-PEKITQCWLGDNDPSFRIID--QNDLPIIGIYPE-Q----------LIHVD---------G-----------SLI-TDW---LPF--------NKM----------------------------------------ESI-------------------------------------------NQVP-LLVGTAAQSIELWP----------------GIEQLRNWTWNEYKKY-VTTSLDSFG---PNLAELTLQLYPI-----PEKSTPESN----TTESELYLANEN--------------------------NLTPEL---------------------LY--ATMVSDIRQNCPVDAVFEQ-------L-SKK--W---------------K--NNIYRYISSGEPSC----------------------P--------IRVYEYESQF-SF--HLWDAIVFFDT-----INQFIH------------------------HPSSDDLNYRDIVQEMVKNFVTNN

>CCE37

QAFFGIPYARQPFGR--FRFT---------------RPEPLT-KLN-DWQLTDDELRRHYKTPPTQARSCPQIDPFG-------------------------------GNFDS----SQSIEDCLRVDLYILDEVFQE---------------------------QRSYDRKYG--------------MGILVEG------FEFKSSVASHL-DLAGGGFKGQSDVGVLAVVHYRLGALGFLTTE---------DD---EGPANLGLWDQSEALRFLKEH--ALNGNIPQVDPQRISLLGFGSGGVSASIH-MLNP-L---NKA--MFSSALVSGGSALGPDASIRDA---RDRAFE-FGQHVGCTS----------SSPRKLINCLRYVD-LQKLVTTG----------------NEANFLFGPV---------------VDVNHTR----D--------VTKAYI-MD----HP---------QRL------------------MERE-------------------NF-------------------------------------------ESIRSVLYGFPEHAASLRYYTQALARA---APLNGRTTAPPDNSIDER----IKYYL-------SPFQSHGDT--------------SRALA---ASVKFLYFRNYT--DET--------LMSNQT-------LFDSLM-------------------------IDALTDYLSVGPVISAAHL-------H-ASK-K--------------LNEQRSGVFIYYRDSEPEL----ATFSSKL-----------PVMS------SASVKYG----T--TMDDLLQFV--------GVK--------------------------GIVPSDQGSEDRLARVWTVVLQDL

>CCE38

CAIRGIPYATA------SRFE---------------RPQALD-E----------TSRKEYHEKKEDTVHCAQTDPFT------------------------------------G--DTIGVENCLVVDLYFPRDR--------------------------------NQAQTRN--------------LVVLLEG------MDFETSLAGF--LSPENSPGETESY-VLAVVHYRTGVFGFMTLQ---------DE---LMPANLGLWDQQMAFKFLRDHASALKV-----NNEFIAL-GFDTGAVALHLH-MLNP-I---SRE--FFKRVLLSGGNALSPGAVTPDA---VTSTMT-LASRLKCDN----------SSMKAVAKCLLKHP-KEDLLKHARA---------------EPALRFASV---------------VDADTSESKSSD-----------AFI------GAPV--------EDL------------------MREK-------------------RT-------------------------------------------GKME-VIIHSYPALGGLNL---------------FARNKVRASKEDDDIKY-IGKFLDMY--G---------------------ERTAETVA---NHIFNQYFKKPG--VK-----------KDIE-------KDPSKF-------------------------IDLLTDFSVGYPVHGLATR-------QIQQY------------------GDATKVESYLLLDPRNGGLKNEAA---------------PSFAAKLKNTKVDQLEGRY-GS--TLDDMLLFV--------GCKKVP-----------------------VAVRNDDKNSREVLGVVSKNAAII

References

1. Claudianos C, Ranson H, Johnson RM, Biswas S, Schuler MA, Berenbaum MR, et al. A deficit of detoxification enzymes: pesticide sensitivity and environmental response in the honeybee. Insect molecular biology. 2006;15(5):615-36. doi: 10.1111/j.1365-2583.2006.00672.x. PubMed PMID: 17069637; PubMed Central PMCID: PMC1761136.

2. Oakeshott JG, Claudianos C, Russell RJ, Robin GC. Carboxyl/cholinesterases: a case study of the evolution of a successful multigene family. BioEssays : news and reviews in molecular, cellular and developmental biology. 1999;21(12):1031-42. doi: 10.1002/(SICI)1521-1878(199912)22:1<1031::AID-BIES7>3.0.CO;2-J. PubMed PMID: 10580988.

3. Zhang J, Li D, Ge P, Yang M, Guo Y, Zhu KY, et al. RNA interference revealed the roles of two carboxylesterase genes in insecticide detoxification in *Locusta migratoria*. Chemosphere. 2013;93(6):1207-15. doi: 10.1016/j.chemosphere.2013.06.081. PubMed PMID: 23899922.
