# Supplementary material for: The Glutathione-S-Transferase, Cytochrome P450 and Carboxyl/Cholinesterase Gene Superfamilies in Predatory Mite Metaseiulus occidentalis
Source: PLoS One. 2016 Jul 28;11(7):e0160009. doi: 10.1371/journal.pone.0160009 (PMC4965064; doi:10.1371/journal.pone.0160009)
Supplement: S1 Table — (DOCX) [file pone.0160009.s005.docx]

**S1 Table.** Details of the *M. occidentalis* GST, CYP, and CCE genes and proteins. Columns are: Gene – the gene and protein name we are assigning; Genes that are clearly pseudogenes end their names with letter “P”; RefSeq – the gene number in the 11,751 RefSeq proteins (prefix is XP_00); GST Class – class assignment of cytosolic GST was based on the results of phylogenetic analysis shown in Figure 1; CYP Clan – clan assignment was based on the results of phylogenetic analysis shown in Figure 3 and results from the cytochrome P450 Nomenclature Committee. * indicates clan assignments only supported by cytochrome P450 Nomenclature Committee; CCE Clade – clade assignment based on the results of phylogenetic analysis in Figure 4; Scaffold – the genome assembly scaffold ID, prefix is scf71800000 (amongst 2210 scaffolds in assembly Mocc1.0); Coordinates – the nucleotide range from the first position of the start codon to the last position of the stop codon in the scaffold; Strand – + is forward and - is reverse; Introns – number of introns; AAs – number of encoded amino acids in the protein; Comments – comments on the Gnomon gene model.

**GSTs**

| Gene | RefSeq | Class | Scaffold | Coordinates | Strand | Introns | AAs | Comments |
| --- | --- | --- | --- | --- | --- | --- | --- | --- |
| GstD1 | 3743487.1 | Delta/Epsilon | 77446 | 1574540-1575703 | + | 4 | 215 | Fine as is |
| GstD2 | 3746787.1 | Delta/Epsilon | 77501 | 1215774-1222708 | + | 3 | 215 | Fine as is |
| GstD3 | 3740940.1 | Delta/Epsilon | 77089 | 51833-53571 | + | 6 | 240 | Fine as is |
| GstM1 | 3745523.1 | Mu | 77484 | 222649-221319 | - | 5 | 239 | Fine as is |
| GstM2 | 3742682.1 | Mu | 77428 | 496711-497379 | + | 0 | 222 | Fine as is |
| GstM3 | 3747949.1 | Mu | 77526 | 938210-938893 | + | 0 | 227 | Fine as is |
| GstM4 | 3737612.1 | Mu | 75579 | 448923-449967 | + | 3 | 230 | Fine as is |
| GstM5 | 3747409.1 | Mu | 77517 | 151331-152819 | + | 3 | 240 | Fine as is |
| GstO1 | 3738156.1 | Omega | 75782 | 189797-186074 | - | 6 | 240 | Partial GST C-terminal domain/ probable pseudogene |
| GstO2 | 3746277.1 | Omega | 77492 | 94273-95696 | + | 5 | 262 | Fine as is |
| GstO3 | 3746278.1 | Omega | 77492 | 94014-92678 | - | 4 | 234 | Fine as is |
| Gst1 | 3744621.1 | Unknown | 77470 | 1059950-1061184 | + | 3 | 161 | Partial GST C-terminal domain/ probable pseudogene |
| GstZ1 | 3744890.1 | Zeta | 77475 | 1471290-1474188 | - | 8 | 247 | Fine as is |
| GstK1 | 3738778.1 | Mitochondrial (Kappa) | 76319 | 70278-71180 | + | 2 | 223 | Fine as is |
| GstK2 | 3738807.1 | Mitochondrial (Kappa) | 76319 | 71747-72655 | + | 2 | 225 | Fine as is |
| PTGSES2 | 3738383.1 | Microsomal | 75896 | 91030-93140 | - | 6 | 383 | Fine as is |

**CYPs**

| Gene | RefSeq | Clan | Scaffold | Coordinates | Strand | Introns | AAs | Comments |
| --- | --- | --- | --- | --- | --- | --- | --- | --- |
| CYP302A1 | 3746072.1 | M | 77487 | 695680-699416 | - | 9 | 535 | First half of model |
| CYP314A1 | 3748577.1 | M | 77533 | 361276-364613 | - | 6 | 482 | Fine as is |
| CYP315A1 | 3743235.1 | M | 77440 | 1100983-1107891 | - | 5 | 549 | Fine as is |
| CYP3012A5 | 3741296.1 | M | 77128 | 619151-621230 | - | 6 | 491 | Fine as is |
| CYP3098A1 | 3745070.1 | M | 77476 | 711967-715620 | + | 7 | 471 | Fine as is |
| CYP307G1 | 3747195.1 | 2 | 77512 | 752021-753887 | - | 4 | 481 | Fine as is |
| CYP3002B1 | 3743832.1 | 2 | 77451 | 165894-168905 | - | 8 | 489 | Fine as is |
| CYP3003B1 | 3745229.1 | 2 | 77478 | 174884-177033 | + | 4 | 494 | Fine as is |
| CYP3099A1 | 3742951.1 | 2 | 77435 | 610573-612071 | - | 3 | 363 | Partial N-terminus/probable pseudogene |
| CYP3099A2 | 3742972.1 | 2 | 77435 | 613821-618192 | - | 5 | 505 | Fine as is |
| CYP3100A1 | 3741017.1 | 2 | 77116 | 627616-635049 | + | 10 | 518 | Fine as is |
| CYP3101A1 | - | 2 | 77522 | 315065-316570 | - | 0 | 500 | New gene model |
| CYP3102A1 | 3747005.1 | 2 | 77511 | 1086884-1089478 | + | 8 | 490 | Fine as is |
| CYP3102A2 | 3747006.1 | 2 | 77511 | 1094296-1097176 | + | 8 | 490 | Fine as is |
| CYP3103A1 | 3742521.1 | 2 | 77404 | 213495-215866 | - | 6 | 503 | Fine as is |
| CYP3104A1 | 3738046.1 | 2* | 75740 | 173422-174945 | - | 0 | 507 | Fine as is |
| CYP3104B1 | 3748194.1 | 2 | 77528 | 84993-86591 | - | 0 | 532 | Fine as is |
| CYP3104C1 | 3748195.1 | 2*** | 77528 | 114398-115939 | + | 0 | 513 | Fine as is |
| CYP3104D1 | 3748197.1 | 2* | 77528 | 143224-144774 | + | 0 | 516 | Fine as is |
| CYP3105A1 | 3738704.1 | 2* | 76281 | 25647-27164 | - | 0 | 505 | Fine as is |
| CYP3106A1 | 3737367.1 | 2* | 75458 | 259240-261219 | + | 1 | 536 | Fine as is |
| CYP3005B1 | - | 3 | 76583 | 1334802-1336509 | + | 2 | 492 | New gene model |
| CYP3005B2 | 3744138.1 | 3 | 77459 | 133702-135397 | - | 2 | 476 | Fine as is |
| CYP3011B1 | 3744549.1 | 3 | 77470 | 229092-231783 | - | 7 | 541 | Fine as is |
| CYP3011B2 | 3744550.1 | 3 | 77470 | 240108-242696 | + | 7 | 542 | Fine as is |
| CYP3011B3 | 3744583.1 | 3 | 77470 | 244697-247361 | + | 6 | 500 | Fine as is |
| CYP3011B4 | 3745699.1 | 3 | 77484 | 1312093-1313700 | + | 0 | 535 | Fine as is |
| CYP3011B5 | 3748678.1 | 3 | 77547 | 6398-8833 | + | 7 | 539 | Fine as is |
| CYP3011B6 | 3743686.1 | 3 | 77450 | 866437-869545 | + | 8 | 534 | Fine as is |
| CYP3011C1 | 3741765.1 | 3 | 77191 | 818695-821442 | - | 9 | 533 | Fine as is |
| CYP3011D1 | 3744236.1 | 3 | 77460 | 1303728-1305287 | + | 0 | 519 | Fine as is |
| CYP3107A1 | 3743440.1 | 3 | 77443 | 273773- 275275 | + | 0 | 500 | Extend N-terminus |
| CYP3107A2 | 3746429.1 | 3 | 77496 | 876847- 878328 | - | 0 | 493 | Fine as is |
| CYP3107A3 | 3742033.1 | 3 | 77244 | 801-1859 | + | 0 | 352 | Extend N-terminus/Partial N-terminus, probable pseudogene |
| CYP3107B1 | 3743386.1 | 3 | 77442 | 576611-577657 | + | 0 | 348 | Partial N-terminus/probable Pseudogene |
| CYP3107C1 | 3738577.1 | 3 | 76006 | 492646-494128 | - | 0 | 494 | Different C-terminus |
| CYP3107D1 | 3737485.1 | 3 | 75554 | 19088-20572 | + | 0 | 494 | Fine as is |
| CYP3107E1 | 3747439.1 | 3 | 77517 | 421902-424872 | - | 1 | 429 | Fine as is |
| CYP3107E2 | 3747435.1 | 3 | 77517 | 391190-392665 | - | 0 | 491 | Fine as is |
| CYP3108A1 | 3740908.1 | 3 | 77088 | 224289-226010 | + | 2 | 514 | Fine as is |
| CYP3109A1 | 3745878.1 | 3 | 77485 | 588334-590036 | + | 2 | 500 | Fine as is |
| CYP3110A1 | 3744817.1 | 3 | 77474 | 815574-817574 | + | 3 | 511 | Fine as is |
| CYP3110B1 | 3743392.1 | 3 | 77442 | 716339-718279 | + | 3 | 509 | Fine as is |
| CYP3111A1 | 3741251.1 | 3 | 77128 | 615731-618733 | + | 10 | 491 | Fine as is |
| CYP4DP3 | 3740735.1 | 4 | 77083 | 188536-192090 | - | 6 | 488 | Fine as is |
| CYP4EN1 | 3740540.1 | 4 | 76951 | 388810-392342 | + | 7 | 511 | Fine as is |
| CYP4EN2 | 3737352.1 | 4 | 75432 | 20255-21793 | + | 0 | 512 | Fine as is |
| CYP4EP1 | 3739201.1 | 4 | 76421 | 97171-98772 | + | 0 | 533 | Extend N-terminus |
| CYP4EP2 | 3738454.1 | 4 | 75950 | 172422-174011 | - | 0 | 529 | Extend N-terminus |
| CYP4EP3 | 3738442.1 | 4 | 75950 | 170072-171649 | - | 0 | 525 | Fine as is |
| CYP4EQ1 | 3746392.1 | 4 | 77495 | 364065-365654 | - | 0 | 529 | Fine as is |
| CYP4ER1 | 3741915.1 | 4 | 77213 | 14380-18038 | - | 9 | 536 | Fine as is |
| CYP4ES1 | 3738212.1 | 4 | 75782 | 514923-517802 | - | 5 | 327 | First half of model/Partial N-terminus, probable pseudogene |
| CYP4ES2 | 3738212.1 | 4 | 75782 | 512344-514925 | - | 7 | 483 | Second half of model |
| CYP4ET1 | 3744205.1 | 4 | 77460 | 452238-458421 | + | 9 | 540 | Second half of model |
| CYP4EU1 | 3744153.1 | 4 | 77460 | 486069-492423 | + | 9 | 522 | Fine as is |
| CYP4EV1 | 3738705.1 | 4 | 76281 | 35424-37031 | - | 0 | 535 | Fine as is |
| CYP4EW1 | 3744207.1 | 4 | 77460 | 495255-497789 | - | 8 | 478 | Fine as is |
| CYP319B1 | 3743204.1 | 4 | 77440 | 369990-372558 | - | 6 | 516 | First half of model |
| CYP319B2 | 3743204.1 | 4 | 77440 | 363904-366268 | - | 6 | 513 | Second half of model |
| CYP319C1 | 3747974.1 | 4 | 77526 | 226726-228704 | - | 5 | 354 | Partial C-terminus, probable pseudogene |
| CYP319C2 | 3747975.1 | 4 | 77526 | 235242-238652 | - | 7 | 503 | Fine as is |
| CYP319C3 | 3747976.1 | 4 | 77526 | 240109-242488 | - | 7 | 507 | Fine as is |
| CYP307G2P | 3747196.1 | 2* | 77512 | 755460-755918 | - | 0 | 152 | Pseudogene |
| CYP3106A2P | 3740355.1 | 2* | 76872 | 116117-117501 | - | 2 | 342 | Pseudogene |
| CYP3107A3P | 3737228.1 | 3* | 75411 | 3368-4466 | + | 1 | 350 | Pseudogene |
| CYP3107A4P | - | 3* | 77496 | 863943-865043 | - | 2 | 315 | Pseudogene |
| CYP3107A5P | 3743371.1 | 3* | 77442 | 304368-307876 | + | 2 | 254 | Pseudogene |
| CYP3107A6P | - | 3* | 77515 | 3534-3833 | + | 0 | 99 | Pseudogene |
| CYP3107A7P | 3746475.1 | 3* | 77496 | 864456-865043 | - | 0 | 195 | Pseudogene |
| CYP3112A1P | 3739531.1 | 3* | 76543 | 649856-651106 | - | 3 | 225 | Pseudogene |
| CYP3113A1P | 3745726.1 | 3* | 77484 | 1739378-1740868 | - | 0 | 496 | Lacks heme-binding motif/pseudogene |
| CYP3113A2P | - | 3* | 77484 | 1734609-1736013 | - | 1 | 430 | Lacks heme-binding motif/pseudogene |
| CYP3114A1P | 3738840.1 | 3* | 76319 | 957478-958170 | + | 0 | 230 | Pseudogene |
| CYP319C4P | 3747973.1 | 4* | 77526 | 219118-222823 | - | 6 | 431 | Pseudogene |
|  |  |  |  |  |  |  |  |  |

**CCEs**

| Gene | RefSeq | CLADE | Scaffold | Coordinates | Strand | Introns | AAs | Comments |
| --- | --- | --- | --- | --- | --- | --- | --- | --- |
| **CCE1** | 3742300.1 | J’’ | 77360 | 213806-216010 | - | 2 | 557 | Different C-terminus |
| **CCE2** | 3742299.1 | J’’ | 77360 | 210742-212474 | - | 1 | 559 | Multiple changes |
| **CCE3** | 3744359.1 | J’’ | 77464 | 912090-915006 | + | 1 | 540 | Fine as is |
| **CCE4** | 3744419.1 | J’’ | 77464 | 907069-908803 | + | 1 | 539 | Fine as is |
| CCE5 | 3740650.1 | J’’ | 77073 | 223531-225654 | - | 1 | 553 | Fine as is |
| **CCE6** | 3740671.1 | J’’ | 77073 | 221504-223284 | - | 1 | 558 | Fine as is |
| **CCE7** | 3740670.1 | J’’ | 77073 | 217525-219431 | - | 1 | 536 | Fine as is |
| **CCE8** | 3745536.1 | J’’ | 77484 | 806716-808634 | - | 1 | 546 | Fine as is |
| **CCE9** | 3747077.1 | J’’ | 77511 | 1163053-1164676 | + | 0 | 533 | Fine as is |
| **CCE10** | 3744142.1 | J’’ | 77459 | 222899-224685 | - | 1 | 547 | Fine as is |
| **CCE11** | 3741940.1 | J’’ | 77213 | 526056-527672 | + | 0 | 538 | Different C-terminus |
| **CCE12** | 3744189.1 | J’’ | 77460 | 58866-60632 | + | 0 | 587 | Fine as is |
| **CCE13** | 3742834.1 | J’’ | 77431 | 122549-124198 | + | 0 | 548 | Fine as is |
| CCE14 | 3738421.1 | J’’ | 75933 | 21811 -23076 | + | 0 | 420 | Partial esterase domain/probable pseudogene |
| CCE15 | 3737960.1 | J’’ | 75644 | 2246-3554 | + | 1 | 419 | Partial esterase domain/probable pseudogene |
| **CCE16** | - | J | 76557 | 2919-6150 | - | 5 | 657 | New gene model |
| **CCE17** | 3739584.1 | J’ | 76543 | 641002-647248 | + | 2 | 636 | Fine as is |
| **CCE18** | 3745369.1 | J’ | 77481 | 5314-9605 | - | 12 | 662 | Fine as is |
| **CCE19** | 3739938.1 | J’ | 76600 | 133173-134894 | + | 1 | 547 | Merge models |
| **CCE20** | 3744479.1 | J’ | 77466 | 482613-484658 | + | 1 | 640 | Fine as is |
| **CCE21** | - | J’ | 77191 | 401044-402549 | - | 0 | 500 | New gene model |
| CCE22 | 3746194.1 | J’ | 77488 | 628871-631856 | + | 4 | 595 | Fine as is |
| **CCE23** | 3741749.1 | J’ | 77191 | 407207-408733 | - | 0 | 507 | Different C-terminus |
| **CCE24** | - | J’ | 77191 | 396814-398418 | - | 0 | 533 | New gene model |
| **CCE25** | 3748301.1 | J’ | 77529 | 1553493-1567933 | + | 6 | 640 | Fine as is |
| **CCE26** | 3743019.1 | J’ | 77436 | 339266-343057 | - | 16 | 644 | Fine as is |
| **CCE27** | 3738701.1 | J’ | 76281 | 7237-9667 | - | 5 | 605 | Fine as is |
| CCE28 | 3741414.1 | J’ | 77167 | 182409-185323 | - | 2 | 528 | First half of model |
| **CCE29** | 3747509.1 | J’ | 77522 | 128490-137634 | - | 12 | 607 | Fine as is |
| CCE30 | 3747841.1 | J’ | 77524 | 2596526-2599323 | - | 8 | 541 | Fine as is |
| CCE31 | 3739668.1 | J’ | 76574 | 225670-231427 | - | 9 | 573 | Merge models |
| **CCE32** | 3742457.1 | J’ | 77384 | 209402-212050 | - | 5 | 577 | Fine as is |
| CCE33 | 3745863.1 | J’ | 77485 | 489110 -491071 | + | 3 | 521 | Second half of model |
| CCE34 | 3743644.1 | J’ | 77448 | 865132-867014 | - | 1 | 568 | Fine as is |
| CCE35 | 3746193.1 | J’ | 77488 | 626404-627804 | + | 0 | 465 | Partial esterase domain/probable pseudogene |
| CCE36 | 3743198.1 | Undetermined | 77440 | 21879-30322 | + | 14 | 657 | Fine as is |
| CCE37 | 3748221.1 | Undetermined | 77528 | 643681-646116 | + | 0 | 662 | Fine as is |
| CCE38 | 3748220.1 | Undetermined | 77528 | 638318-640516 | - | 0 | 665 | Fine as is |
| CCE39 | 3747068.1 | K | 77511 | 1123005-1129298 | + | 9 | 771 | Fine as is |
| CCE40 | 3740840.1 | L | 77086 | 1032770-1053402 | - | 9 | 817 | Fine as is |
| CCE41 | 3741692.1 | L | 77190 | 323301-339567 | - | 13 | 901 | Fine as is |
| CCE42 | 3742198.1 | L | 77293 | 26631-48713 | - | 13 | 934 | Fine as is |
| CCE43 | 3740818.1 | L | 77086 | 423349-433938 | - | 12 | 927 | Fine as is |
| CCE44 | 3747809.1 | L | 77524 | 2041242-2051763 | + | 10 | 853 | Fine as is |
| CCE45P | 3746248.1 | NA | 77488 | 1579037-1588811 | - | 6 | 231 | Pseudogene |

CCEs highlighted in bold contain a catalytic triad (Ser-Glu (Asp)-His) and the nucleophilic elbow surrounding the active-site serine residue (GXSXG) [1-3]. For details, see S5 Fig.

**References**

1. Claudianos C, Ranson H, Johnson RM, Biswas S, Schuler MA, Berenbaum MR, et al. A deficit of detoxification enzymes: pesticide sensitivity and environmental response in the honeybee. Insect molecular biology. 2006;15(5):615-36. doi: 10.1111/j.1365-2583.2006.00672.x. PubMed PMID: 17069637; PubMed Central PMCID: PMC1761136.

2. Oakeshott JG, Claudianos C, Russell RJ, Robin GC. Carboxyl/cholinesterases: a case study of the evolution of a successful multigene family. BioEssays : news and reviews in molecular, cellular and developmental biology. 1999;21(12):1031-42. doi: 10.1002/(SICI)1521-1878(199912)22:1<1031::AID-BIES7>3.0.CO;2-J. PubMed PMID: 10580988.

3. Zhang J, Li D, Ge P, Yang M, Guo Y, Zhu KY, et al. RNA interference revealed the roles of two carboxylesterase genes in insecticide detoxification in *Locusta migratoria*. Chemosphere. 2013;93(6):1207-15. doi: 10.1016/j.chemosphere.2013.06.081. PubMed PMID: 23899922.
