# Supplementary material for: The Glutathione-S-Transferase, Cytochrome P450 and Carboxyl/Cholinesterase Gene Superfamilies in Predatory Mite Metaseiulus occidentalis
Source: PLoS One. 2016 Jul 28;11(7):e0160009. doi: 10.1371/journal.pone.0160009 (PMC4965064; doi:10.1371/journal.pone.0160009)
Supplement: S2 Table — (DOCX) [file pone.0160009.s006.docx]

**S2 Table.** Gene ID and accession numbers of the GST sequences from several arthropods used for phylogenetic analyses.

*D. melanogaster*

| FlyBase annotation  symbol | Name in tree |
| --- | --- |
| CG10045 | Dm_GstD1 |
| CG4423 | Dm_GstD6 |
| CG18548 | Dm_GstD10 |
| CG5164 | Dm_GstE1 |
| CG17530 | Dm_GstE6 |
| CG17522 | Dm_GstE10 |
| CG6662 | Dm_GstO1 |
| CG6673 | Dm_GstO2 |
| CG8938 | Dm_GstS1 |
| CG30000 | Dm_GstT1 |
| CG30005 | Dm_GstT2 |

FlyBase: http://flybase.org/

*A. mellifera*

| BeeBase identifier | Name in tree |
| --- | --- |
| GB50265 | Am_GstD1 |
| GB44803 | Am_GstO1 |
| GB48905 | Am_GstS1 |
| GB49545 | Am_GstS4 |
| GB42961 | Am_GstT4 |

BeeBase :http://hymenopteragenome.org/beebase/

*A. gambiae*

| GenBank accession | Name in tree |
| --- | --- |
| AAC79992.1 | Ag_GstD1 |
| AAM53606.1 | Ag_GstD5 |
| AAM53609.1 | Ag_GstD11 |
| XP_319969.1 | Ag_GstE1 |
| XP_319966.1 | Ag_GstE5 |
| XP_319963.1 | Ag_GstE8 |
| XP_315763.2 | Ag_GstO1 |
| P46428.4 | Ag_GstS |
| XP_311299.1 | Ag_GstT1 |
| XP_316865.2 | Ag_GstT2 |

*T. urticae*

| OrcAE gene ID | Name in tree |
| --- | --- |
| tetur01g02230 | Tu_GstD1 |
| tetur01g02480 | Tu_GstD3 |
| tetur03g07920 | Tu_GstD6 |
| tetur26g02802 | Tu_GstD10 |
| tetur31g01390 | Tu_GstD16 |
| tetur03g09230 | Tu_GstM1 |
| tetur05g05240 | Tu_GstM7 |
| tetur05g05300 | Tu_GstM12 |
| tetur01g02320 | Tu_GstO1 |
| tetur12g03900 | Tu_GstO2 |

OrcAE: http://bioinformatics.psb.ugent.be/orcae/overview/Tetur

*I. scapularis*

| VectorBase ID | Name in tree |
| --- | --- |
| ISCW005803 | Is_GstD2 |
| ISCW024224 | Is_GstD4 |
| ISCW005804 | Is_GstE1 |
| ISCW007012 | Is_GstE3 |
| ISCW004523 | Is_GstM1 |
| ISCW017235 | Is_GstM7 |
| ISCW022157 | Is_GstM12 |
| ISCW003939 | Is_GstO1 |

VectorBase: https://www.vectorbase.org/
