# Supplementary material for: The Glutathione-S-Transferase, Cytochrome P450 and Carboxyl/Cholinesterase Gene Superfamilies in Predatory Mite Metaseiulus occidentalis
Source: PLoS One. 2016 Jul 28;11(7):e0160009. doi: 10.1371/journal.pone.0160009 (PMC4965064; doi:10.1371/journal.pone.0160009)
Supplement: S3 Table — (DOCX) [file pone.0160009.s007.docx]

**S3 Table.** Gene ID of the CYP sequences of *D. melanogaster*, *A. mellifera* and *T. urticae* used for phylogenetic analyses.

*D. melanogaster*

| FlyBase symbol | Name in tree |
| --- | --- |
| CG6816 | Dm_CYP18A1 |
| CG6578 | Dm_CYP306A1 |
| CG4163 | Dm_CYP303A1 |
| CG8733 | Dm_CYP305A1 |
| CG7241 | Dm_CYP304A1 |
| CG10594 | Dm_CYP307A1 |
| CG9438 | Dm_CYP6A2 |
| CG8453 | Dm_CYP6G1 |
| CG4485 | Dm_CYP9B1 |
| CG3616 | Dm_CYP9C1 |
| CG1488 | Dm_CYP311A1 |
| CG8587 | Dm_CYP301A1 |
| CG13478 | Dm_CYP314A1 |
| CG14728 | Dm_CYP315A1 |
| CG18377 | Dm_CYP49A1 |

*A. mellifera*

| BeeBase identifier | Name in tree |
| --- | --- |
| GB54765 | Am_CYP18A1 |
| GB47752 | Am_CYP303A1 |
| GB47885 | Am_CYP304A1 |
| GB48175 | Am_CYP305A1 |
| GB48993 | Am_CYP6A8 |
| GB46814 | Am_CYP6K1 |
| GB44513 | Am_CYP4C1 |
| GB51356 | Am_CYP4G11 |
| GB42898 | Am_CYP12A5 |
| GB46062 | Am_CYP49A1 |
| GB46015 | Am_CYP301A1 |
| GB47901 | Am_CYP302A1 |
| GB45651 | Am_CYP314A1 |
| GB53709 | Am_CYP315A1 |

*T. urticae*

| OrcAE gene ID | Name in tree |
| --- | --- |
| tetur10g03900 | Tu_CYP307A1 |
| tetur23g00260 | Tu_CYP392D1 |
| tetur06g04520 | Tu_CYP392A16 |
| tetur20g03200 | Tu_CYP392B1 |
| tetur03g01560 | Tu_CYP382A1 |
| tetur38g00660 | Tu_CYP384A1 |
| tetur07g05500 | Tu_CYP385A1 |
| tetur08g06170 | Tu_CYP387A1 |
| tetur36g00920 | Tu_CYP391A1 |
| tetur01g04440 | Tu_CYP406A1 |
| tetur03g03020 | Tu_CYP314A1 |
| tetur06g05620 | Tu_CYP315A1 |
| tetur13g02850 | Tu_CYP381A1 |

OrcAE: http://bioinformatics.psb.ugent.be/orcae/search/in/Tetur/current
