# Supplementary material for: The Glutathione-S-Transferase, Cytochrome P450 and Carboxyl/Cholinesterase Gene Superfamilies in Predatory Mite Metaseiulus occidentalis
Source: PLoS One. 2016 Jul 28;11(7):e0160009. doi: 10.1371/journal.pone.0160009 (PMC4965064; doi:10.1371/journal.pone.0160009)
Supplement: S4 Table — (DOCX) [file pone.0160009.s008.docx]

**S4 Table.** Gene ID of the CCE sequences of *D. melanogaster,* *A. mellifera* and *T. urticae* used for phylogenetic analysis.

*D. melanogaster*

| FlyBase symbol | Name in tree |
| --- | --- |
| CG17907 | Dm_CG17907 |
| CG1121 | Dm_CG1121 |
| CG2505 | Dm_CG2505 |
| CG8425 | Dm_CG8425 |
| CG17148 | Dm_CG17148 |
| CG6917 | Dm_CG6917 |
| CG5397 | Dm_CG5397 |
| CG9280 | Dm_CG9280 |
| CG9287 | Dm_CG9287 |
| CG9289 | Dm_CG9289 |
| CG3903 | Dm_CG3903 |
| CG31146 | Dm_CG31146 |
| CG13772 | Dm_CG13772 |
| CG12869 | Dm_CG12869 |
| CG9704 | Dm_CG9704 |

*A. mellifera*

| Beebase identifier | Name in tree |
| --- | --- |
| GB18414 | Am_GB18414 |
| GB14873 | Am_GB14873 |
| GB11064 | Am_GB11064 |
| GB16342 | Am_GB16342 |
| GB10820 | Am_GB10820 |
| GB15327 | Am_GB15327 |
| GB12309 | Am_GB12309 |
| GB18290 | Am_GB18290 |
| GB18836 | Am_GB18836 |
| GB18720 | Am_GB18720 |
| GB10066 | Am_GB10066 |
| GB13939 | Am_GB13939 |
| GB19830 | Am_GB19830 |

*T. urticae*

| OrcAE gene ID | Name in tree |
| --- | --- |
| tetur19g00850 | Tu_19g00850 |
| tetur04g06380 | Tu_04g06380 |
| tetur02g06930 | Tu_02g06930 |
| tetur30g01560 | Tu_30g01560 |
| tetur04g08480 | Tu_04g08480 |
| tetur01g14090 | Tu_01g14090 |
| tetur01g10830 | Tu_01g10830 |
| tetur01g10760 | Tu_01g10760 |
| tetur11g05770 | Tu_11g05770 |
| tetur29g00970 | Tu_29g00970 |

OrcAE: http://bioinformatics.psb.ugent.be/orcae/search/in/Tetur/current
